# Dual-Modal Magnetic Resonance/Fluorescent Zinc Probes for Pancreatic β-Cell Mass Imaging

**DOI:** 10.1002/chem.201406008

**Published:** 2015-03-03

**Authors:** Graeme J Stasiuk, Florencia Minuzzi, Myra Sae-Heng, Charlotte Rivas, Hans-Paul Juretschke, Lorenzo Piemonti, Peter R Allegrini, Didier Laurent, Andrew R Duckworth, Andrew Beeby, Guy A Rutter, Nicholas J Long

**Affiliations:** [a] Department of Chemistry, Imperial College LondonSouth Kensington Campus, London SW7 2AZ (UK); [b] Section of Cell Biology and Functional Genomics, Division of Diabetes, Endocrinology and Metabolism, Department of MedicineImperial College London, Hammersmith Hospital, London W12 0NN (UK); [c] Sanofi-Aventis Deutschland GmbH, R&D DSAR/BiomakersBiom & Biol Ass, FF, Industriepark Hoechst, Building H825, 65926 Frankfurt (Germany); [d] Diabetes Research Institute, IRCCS San Raffaele Scientific InstituteVia Olgettina 60, 20132 Milano (Italy); [e] Novartis Pharma AG, Fabrikstrasse28-3.04, 4002 Basel (Switzerland); [f] Novartis Institute for Biomedical Research, Fabrikstrasse10-2.40.4, 4056, Basel (Switzerland); [g] Department of Chemistry, Durham University, South RoadDurham, DH1 3LE (UK)

**Keywords:** diabetes, fluorescence, imaging agents, lanthanides, zinc

## Abstract

Despite the contribution of changes in pancreatic β-cell mass to the development of all forms of diabetes mellitus, few robust approaches currently exist to monitor these changes prospectively in vivo. Although magnetic-resonance imaging (MRI) provides a potentially useful technique, targeting MRI-active probes to the β cell has proved challenging. Zinc ions are highly concentrated in the secretory granule, but they are relatively less abundant in the exocrine pancreas and in other tissues. We have therefore developed functional dual-modal probes based on transition-metal chelates capable of binding zinc. The first of these, Gd**⋅1**, binds Zn^II^ directly by means of an amidoquinoline moiety (AQA), thus causing a large ratiometric Stokes shift in the fluorescence from *λ*_em_=410 to 500 nm with an increase in relaxivity from *r*_1_=4.2 up to 4.9 mM^−1^ s^−1^. The probe is efficiently accumulated into secretory granules in β-cell-derived lines and isolated islets, but more poorly by non-endocrine cells, and leads to a reduction in *T*_1_ in human islets. In vivo murine studies of Gd**⋅1** have shown accumulation of the probe in the pancreas with increased signal intensity over 140 minutes.

## Introduction

Changes in pancreatic β-cell mass contribute to the development of both type 1 and type 2 diabetes (T1D and T2D, respectively), diseases that currently affect more than 340 million individuals worldwide[1] and whose incidence in adults is projected to increase by approximately 20 % by 2030. Whereas immune cell-mediated mechanisms lead to near-complete elimination of β cells in T1D, both the extent of the loss (estimates range from approximately 35[2] to 60 %)[3] and the underlying mechanisms are less well established for T2D.[4] Nonetheless, these are likely to involve the interplay of genetic and environmental factors, including obesity. A critical roadblock in understanding both disease mechanisms and progression is, however, the absence of robust approaches to following changes in β-cell mass prospectively in vivo.

Compared with other modalities, magnetic resonance (MR) imaging has excellent anatomical resolution but suffers on the molecular scale from low intrinsic sensitivity. Thus, to produce a detectable change in water signal intensity, a relatively high concentration of contrast agent (0.01–0.1 mmol kg^−1^) is required. This creates problems when imaging at the molecular level, as the most interesting (i.e., cell-type-specific) targets are likely to be present at much lower concentrations (pM–nM). Dual-modal imaging potentially offers important advantages. Here, the goal is to overlay images obtained by different techniques, thereby giving better image resolution and co-validation of the accumulation of targeted contrast agents (for example, in animal models) or probes at a specific site.[5]

Over the last fifteen years, research into contrast agents that respond to different concentrations of metal ions, such as Ca^II^, Fe^II/III^ and Zn^II^, has given an indication of disease states and become a key area of interest.[6] These probes can be split into three classes: fluorescent, MRI, and more recently, dual-modal imaging agents. There has been considerable interest in zinc sensing for disease states such as diabetes. For example, a plethora of Zn^II^-sensing fluorescent probes based on a variety of organic fluorophores such as 4,4-difluoro-4-bora-3a,4a- diaza-*s*-indacene (bodipy)[7] and rhodamine,[8] modified to include Zn^II^ chelating units—that is, dipicolylamine (DPA)—have previously been described and examined in depth by Nagano and co-workers.[9] The quinoline-based AQA probe developed by Zhang et al.[10] is an organic fluorophore and Zn^II^ chelator that shows excellent ratiometric fluorescence properties upon zinc binding. Other examples of organic zinc fluorescent probes have also been developed by Lippard and co-workers.[11]

MRI-based Zn^II^ probes were first developed by Nagano and co-workers, who attached two DPA units to Gd–DTPA (DTPA=diethylene triamine pentaacetic acid).[12] This approach to imaging zinc was also successfully employed by Sherry and co-workers, who used a probe based around the DOTA motif, with two DPA Zn^II^ binding units, to image zinc release.[13] Similarly, Pope and co-workers have developed an Eu^III^ 1,4,7,10-tetraazacyclododecane-1,4,7-trisacetic acid (DO3A)-based Zn^II^ sensor,[14] whereas Meade and co-workers have described a Zn^II^ sensing Gd–DOTA-based (DOTA=1,4,7,10-tetraazacyclododecane-1,4,7,10-tetraacetic acid) chelate that shows a strong zinc-dependent increase in relaxivity (*r*_1_=2.3 mM^−1^ s^−1^ in the absence of Zn^II^ and 5.1 mM^−1^ s^−1^ in its presence).[15] Parker and co-workers have developed DOTA-based Zn^II^-responsive MRI contrast agents with Gd^III^ that show a 30–40 % increase in *r*_1_, and fluorescent probes with Eu^III^, which show an increase in fluorescence intensity upon metal chelation.[16]

The development of dual-modality MR/fluorescent zinc-sensing imaging agents represents a particularly attractive approach. In principle, this allows the distribution of the probe in cells and tissues to be explored in vitro with high resolution and sensitivity prior to in vivo studies using MRI. Thus, Luo et al. have recently described a DOTA-based chelate attached to a quinoline derivative that binds Zn^II^ with a relaxivity of 3.8 mM^−1^ s^−1^, a value that increases upon addition of half an equivalent of Zn^II^ to 5.9 mM^−1^ s^−1^ and decreases back to 5.2 mM^−1^ s^−1^ (at 23 MHz). The fluorescence emission of this probe also shows a similar increase at up to 0.5 equivalents of Zn^II^ but decreases in the same manner as the relaxivity.[17] Nagano et al. also showed that a DTPA–quinoline DPA conjugate can be used as a dual MR/fluorescent sensor with Gd^III^ and a dual fluorescent sensor with Eu^III^ for Zn^II^, with the latter showing an increase in fluorescence intensity with Zn^II^.[18] Pope and co-workers have shown that a dual fluorescent Zn^II^ sensor based on DOTA with quinoline and isoquinoline, and which incorporates Eu^III^ and Yb^III^, shows increasing fluorescence upon Zn^II^ chelation.[19] However, none of these previously described dual-modal/bimodal probes exhibit ratiometric fluorescence changes in response to Zn^II^ binding.

Given the high concentration of Zn^II^ ions in insulin granules,[20] a substantial proportion of which can potentially be unbound, and the relative scarcity of zinc ions throughout the rest of the pancreas,[21] we hypothesised that MRI-active agents capable of binding Zn^II^ might be useful as a means of quantifying β-cell mass in vivo. Specifically, we used DOTA- and DO3A-based macrocycles, which are excellent chelating agents for MRI-active metal ions, and functionalised them to allow both Zn^II^ binding and the incorporation of fluorescent sensors. Coordination with gadolinium or other MRI-active transition-metal ions yields novel dual-modal, MR/fluorescent zinc-sensing probes. We show here that these are tropic for pancreatic β-cell granules in vitro and indeed we report the first dual-modal imaging probe to accumulate within granules. Moreover, the properties (cell permeability and charge) and zinc-dependent fluorescence of these probes allow them to serve as sensors for free Zn^II^ within sub-compartments of single cells.

## Results and Discussion

Herein we describe dual-modal MR/fluorescent contrast agents that show specific binding to Zn^II^ as tools to image β-cell mass and function in diabetes. DO3A is proven to be an excellent chelate that can be functionalised for many biological imaging purposes.[22] This macrocycle has been modified with the organic fluorophore AQA, which has a built-in Zn^II^ sensing motif, in combination with Gd^III^ to yield a novel dual-modal Zn^II^ sensing probe. Compound Gd**⋅1** shows ratiometric fluorescence changes with a large Stokes shift from *λ*=410 to 500 nm upon zinc chelation and *K*_d_=22 μM (pH 7.4). It should be noted that this wavelength is not ideal for imaging in vivo but is useful for these proof-of-principle studies. Compound Gd**⋅1** has an *r*_1_=4.2 mM^−1^ s^−1^ (9.4 T), which increased as high as 6.6 mM^−1^ s^−1^ (9.4 T) upon Zn^II^ binding. When examined in β-cell lines, Gd**⋅1** (and Eu**⋅1**) also showed localisation to secretory granules identified by co-expression of granule-resident protein phogrin[23] and relatively poor uptake into non-β cells.

**Scheme 1:**
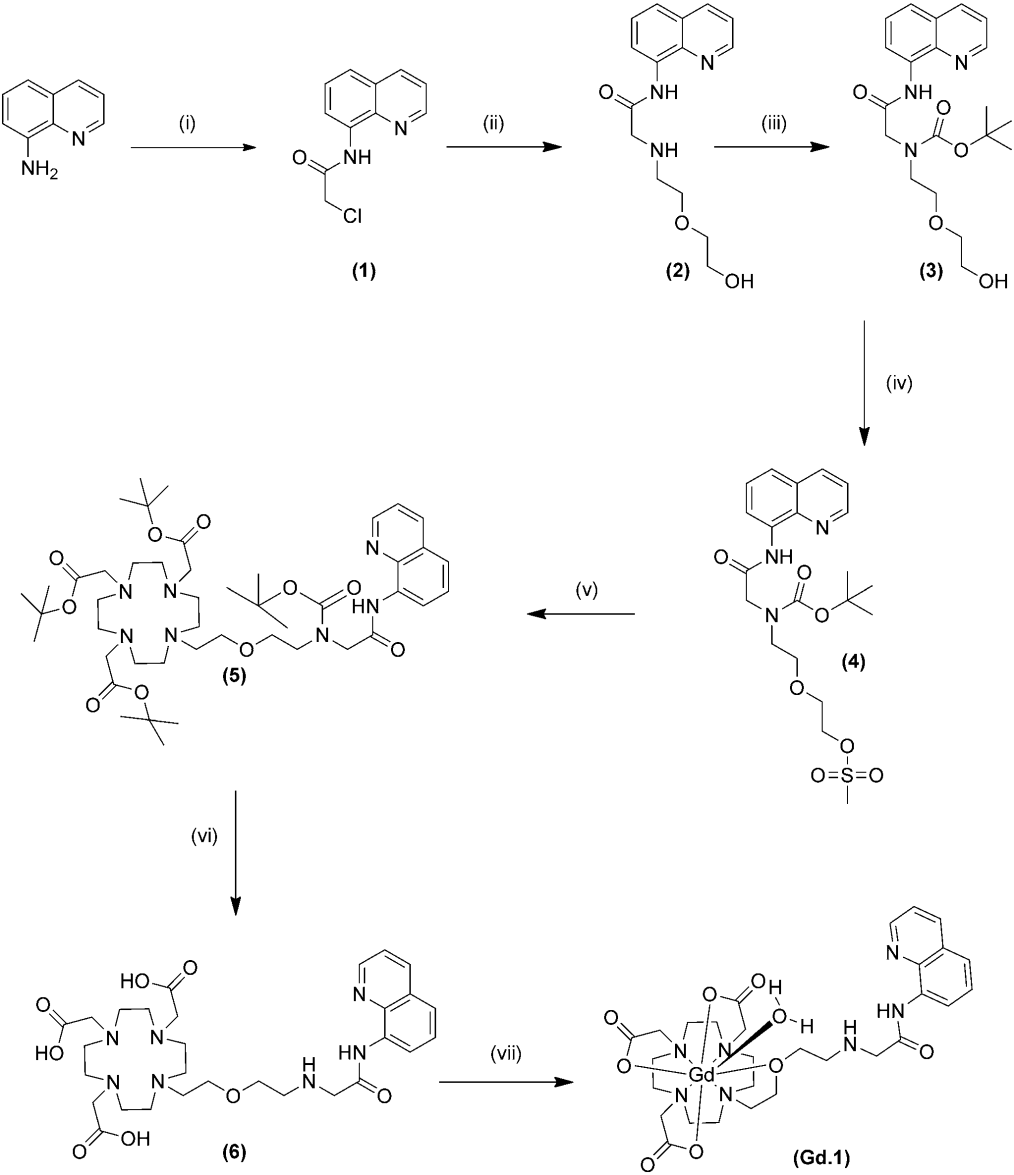
Formation of Gd⋅1. i) Chloroacetyl chloroacetate, pyridine/CHCl_3_ (79%). ii) 2-(2-Aminoethoxy)ethanol, DIPEA, KI, MeCN (90%). iii) Boc anhydride, pyridine, THF (90%). iv) Methane sulfonyl chloride, DCM, triethylamine (90%). v) *t*Bu DO_3_A, K_2_CO_3_, DCM, (77%). vi) TFA, DCM (>99%). vii) GdCl_3_⋅6 H_2_O, H_2_O, pH 5.5 (91%).

The AQA-DO3A conjugate **6** was synthesised in six steps from the organic dye 8-aminoquinoline and cyclen. The first two steps synthesised the AQA dye (**2**) as described by Zhang et al.[10] (Scheme 1 and Figure S1 of the Supporting Information). The next step conjugated the above organic Zn^II^ sensor to the cyclen-based chelate DO3A to form the dual-modal organic framework.[24] Protection of the free amine of the AQA group with di-*tert*-butyl dicarbonate is a facile reaction and gave **3** in 90 % yield, thereby preventing the intramolecular cyclisation of the AQA organic fluorophores under basic conditions. Compound **3** was treated with mesyl chloride to activate the OH of the protected AQA fluorophore to give **4** in a 90 % yield. The mesyl AQA was treated with *tert*-butyl DO3A without side reactions to yield **5** in 77 % yield. Compound **6** was formed by deprotecting the *tert*-butyl esters with TFA in a quantitative yield. The DO3A–AQA conjugate can easily complex lanthanide metal ions: Gd^III^ for a Zn^II^-sensing dual-modal MR/fluorescent probe, and Eu^III^ for a Zn^II^-sensing dual-fluorescent probe. Compound Gd**⋅1** was synthesised from **6** and GdCl_3_**⋅**6 H_2_O in 92 % yield; Eu**⋅1** was synthesised from **6** and the corresponding europium chloride salt in 95 % yield.

**Figure 1:**
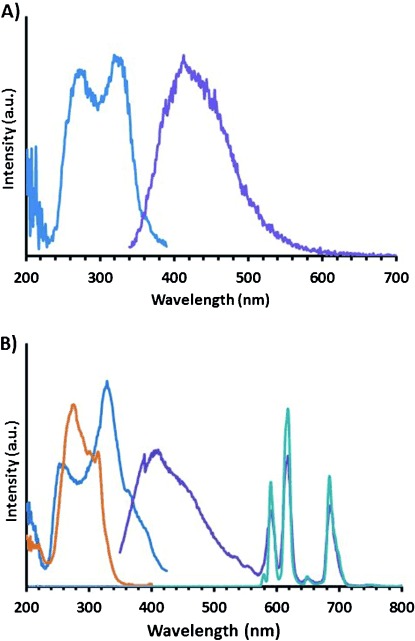
A) Fluorescence spectra for Gd⋅1 showing the excitation spectrum (dark blue, monitored at *λ*=410 nm) and the emission spectrum (purple, excited at *λ*=320 nm). B) Luminescence spectra for Eu⋅1 showing the excitation spectrum with 0 ms delay (dark blue, monitored at *λ*=616, 410 nm), excitation spectrum with 0.1 ms delay (orange, monitored at *λ*=616 nm), emission spectrum with 0 ms delay (purple, excited at *λ*=350 nm) and emission spectrum with 0.1 ms delay (light blue, excited at *λ*=350 nm) (pH 7.4, 298 K).

Compound Gd**⋅1** shows a maximum emission peak at *λ*=410 nm in the fluorescence spectrum (Figure 1a). This is the standard emission for the AQA moiety and can be excited at *λ*=280 and 320 nm.[10] Relaxation measurements at 400 MHz (9.4 T) on Gd**⋅1** show an *r*_1_=4.2 mM^−1^ s^−1^, which suggests a hydration state (*q*) of one, much like the ‘gold standard’ MRI contrast agent Gd**⋅DOTA**, which has an *r*_1_=4.2 mM^−1^ s^−1^.[22,25] This is unexpected as the arrangement of the coordinating ligands suggests that two water molecules should bind. There might be two possible explanations for this. Either the AQA arm bulk restricts access of one water from binding to the Gd^III^ metal centres, as observed by Mishra et al. with a fluorescein isothiocyanate (FITC) moiety conjugated to a DO3A motif with an *r*_1_ of 5.36 mM^−1^ s^−1^;[26] or the oxygen in the pendant AQA arm might be bound to the lanthanide metal centre to give an eight-coordinate structure with one bound water ligand. The *q* of 1 was confirmed by the lifetime studies of Eu**⋅1** (Table 1), and the proton NMR spectrum of Eu**⋅1** (Figure S26 in the Supporting Information) suggests that the oxygen of the AQA side arm is bound to the lanthanide, thereby causing the *q* of 1 rather than the steric hindrance from the AQA moiety; this can be seen by the axial protons around *δ*=25–20 ppm[27] in the square-antiprismatic (SAP) isomer and *δ*=15–12 ppm in the twisted square-antiprismatic (TSAP) isomer at room temperature. Figure 1 shows the excitation and emission spectra for Gd**⋅1** (Figure 1a) and Eu**⋅1** (Figure 1b). There is no difference in the emission spectrum for the AQA moiety at *λ*=410 nm, thus indicating that the coordination of the emissive lanthanide ion does not change the emission from the AQA moiety. When excited at *λ*=260 nm, with a zero millisecond delay, Eu**⋅1** shows both emission from the AQA and Eu^III^ metal at *λ*=410 and 616 nm, respectively (purple, Figure 1B), thus indicating that the AQA moiety acts as an organic fluorophore and an antenna for Eu^III^. The excitation spectra (blue) when monitoring *λ*=410 nm (and *λ*=616 nm, not shown) shows a large peak at *λ*=250 and 350 nm that corresponds to the AQA ligand. When the emission and excitation spectra are taken with a delay of 0.1 ms to eliminate emission from the AQA given that the lifetimes of organic fluorophores are in the microsecond range and lanthanide ions are in the millisecond range, only emission from the Eu^III^ metal (orange and light blue, respectively; Figure 1B) is observed. The excited state of amidoquinoline is approximately 20 800 cm^−1^,[19] which allows for energy transfer from the triplet state of the amidoquinoline to the ^5^D_0_ state of the Eu^III^ metal, that is, approximately 17 250 cm^−1^. The form of the spectra for Eu**⋅1** suggests a hydration state of one (light blue), given that the Δ*J*=1 at 595 nm is of a relatively similar intensity to that of Δ*J*=2 at 616 nm, which suggests a coordination geometry of eight rather than seven, in which case the Δ*J*=1 would be significantly lower than the Δ*J*=2.[27] This confirms the postulation that the oxygen on the AQA side arm binds to the metal centre. Compound Eu**⋅1** provides an unusual dual-fluorescent probe, which has two defined emission wavelengths on two different timescales: the millisecond from the lanthanide ion and the nanosecond from the organic fluorophore.

**Table 1:** Gd⋅1 versus Zn relaxivity (400 MHz) and Eu⋅1 versus Zn hydration state (*q*) (1 mM Ln⋅1, pH 7.4, 298 K)

	Zn [mM]	0	0.5	1	2	5
Gd**⋅1**	*r*_1_ [mM^−1^ s^−1^]	4.2	6.6	5.9	4.6	4.9
Eu**⋅1**	*q*	1.1	1.1	1.1	1.1	1.1

**Figure 2:**
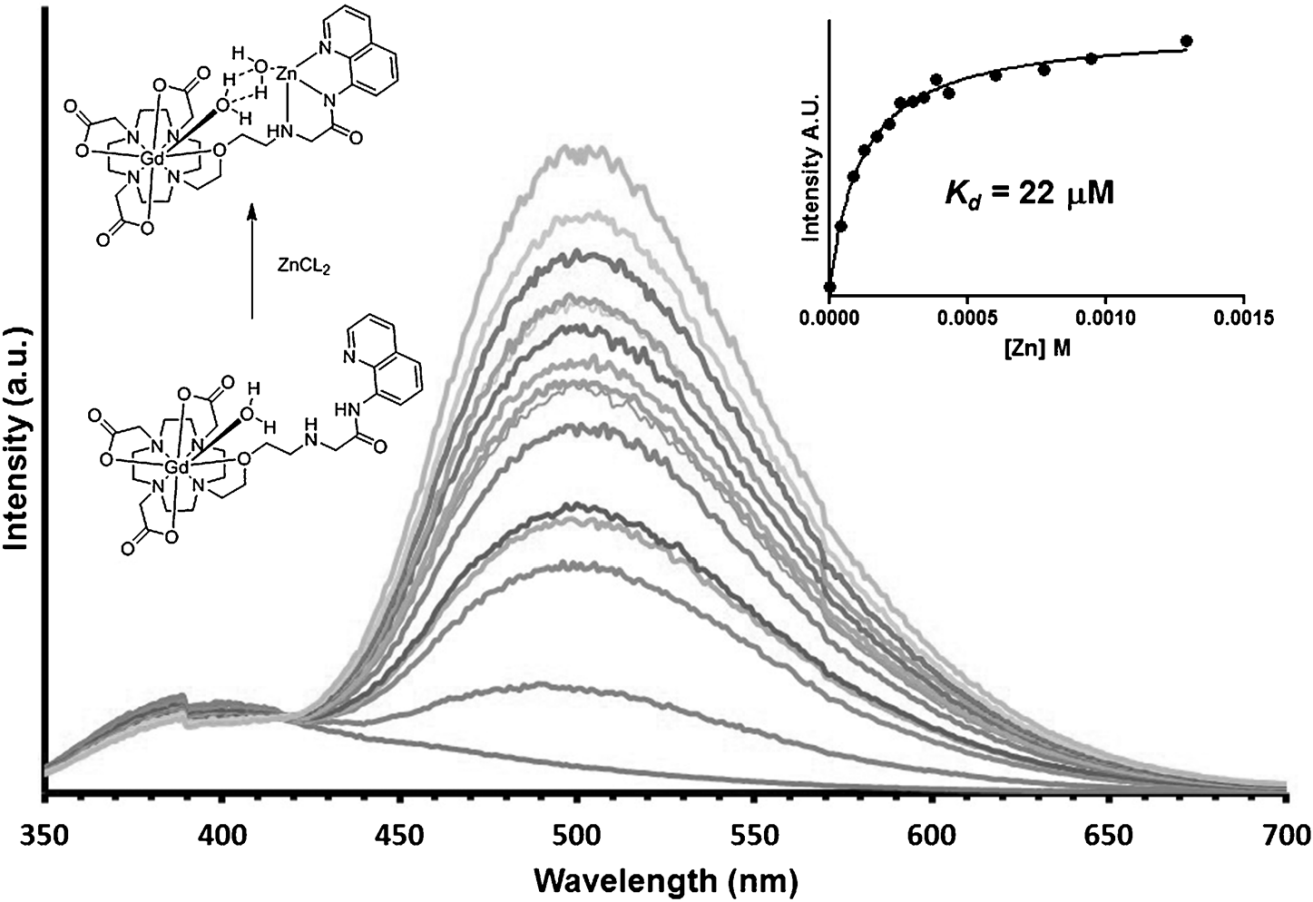
Gd⋅1 versus Zn^II^, fluorescence (1 mM, pH 7.4, 298 K, *λ*=280 nm).

A potential complication of using Zn^II^ as a target for probes to measure β-cell mass is the presence within the secretory granules of Ca^II^ ions, which might conceivably interfere. Compound Gd**⋅1** shows a ratiometric fluorescent response to Zn^II^ (Figure 2), such that upon Zn^II^ chelation there is a large (90 nm) Stokes shift towards the red in emission, from 410 to 500 nm, and a 300 % increase in fluorescence amplitude. These fluorescent properties thus provide the indicator with excellent Zn^II^-sensing properties. By contrast, studies with other metals such as calcium and copper revealed no change in fluorescence upon addition of either ion (Figure S2 in the Supporting Information), thus demonstrating the specificity of Gd**⋅1** as a fluorescent zinc sensor. The UV/Vis titration of Gd**⋅1** with Zn^II^ shows an increase in intensity at 250 nm, whereas the UV/Vis titrations with Ca^II^ show binding to Gd**⋅1** (Figure S3 in the Supporting Information), with no formation of this band at 250 nm.

The relaxivity (*r*_1_) of Gd**⋅1** is significantly changed when binding to zinc (Table 1). Thus, in the absence of Zn^II^, *r*_1_ was 4.2 mM^−1^ s^−1^, thus suggesting a hydration state of one. Upon binding 0.5 equivalents of Zn^II^, the value of *r*_1_ increased to 6.6 mM^−1^ s^−1^. It can therefore be postulated that a metallostar compound is being created with two Gd**⋅1** units coordinating one Zn^II^ molecule, as seen previously in similar compounds.[17] The Eu**⋅1** hydration state remains constant at one, irrespective of the binding of Zn^II^. As the concentration of Zn^II^ increases beyond two equivalents, the relaxivity of Gd**⋅1** drops to *r*_1_=4.9 mM^−1^ s^−1^, still with a hydration state of one, suggesting a one-to-one ratio of Gd**⋅1** to Zn. The binding of Zn^II^ is postulated therefore to increase second-sphere water ordering, with possible hydrogen bonding to a solvated zinc to the outer sphere, thus explaining the increase in *r*_1_ from 4.2 to 4.9 mM^−1^ s^−1^.

The dissociation constant of Gd**⋅1** for zinc at pH 7.4 was measured as 22 μM, a value lower than that of 37 μM for Ca^II^, thus suggesting that binding of either will be possible in cells depending on the relative free concentrations of the two. The selective nature of Gd**⋅1** for Zn^II^ can also be seen in the shift and increased intensity of the emission, which occurs when Gd**⋅1** comes into contact with Zn^II^, but not Ca^II^, thus providing significant advantages as a sensor for Zn^II^. This difference in responses to the two ions can be seen in Figure S4 of the Supporting Information, whereby five equivalents of Ca^II^ to Gd**⋅1** elicit no increase in fluorescence emission. Upon addition of Zn^II^, in contrast, the emission spectrum increases in intensity and shifts to 500 nm after the addition of more than 1 equivalent of Zn^II^. The calculated constant for this interaction matches the difference in the constants of Gd**⋅1** to Zn^II^ and Ca^II^. Thus, Gd**⋅1** shows a ratiometric fluorescent response to Zn^II^ with an increase in relaxivity, and a *K*_d_ potentially in an ideal range for Zn^II^ sensing in β-cell granules, thereby providing the basis for an excellent dual-modal imaging agent. It should be noted that the pH in the granule is approximately 6,[28] for which the *K*_d_ for Zn^II^ to Gd**⋅1** was measured as 111 μM. This has been confirmed by a pH titration using Eu**⋅1** (Figure S34 in the Supporting Information) that shows a p*K*_a_ of 7.35, which suggests that amide deprotonation is vital to ensure tight binding to Zn^II^, therefore at pH 7.4 the affinity is greater than at pH 6 at which a large proportion of the Ln**⋅1** amide would be protonated.

**Figure 3:**
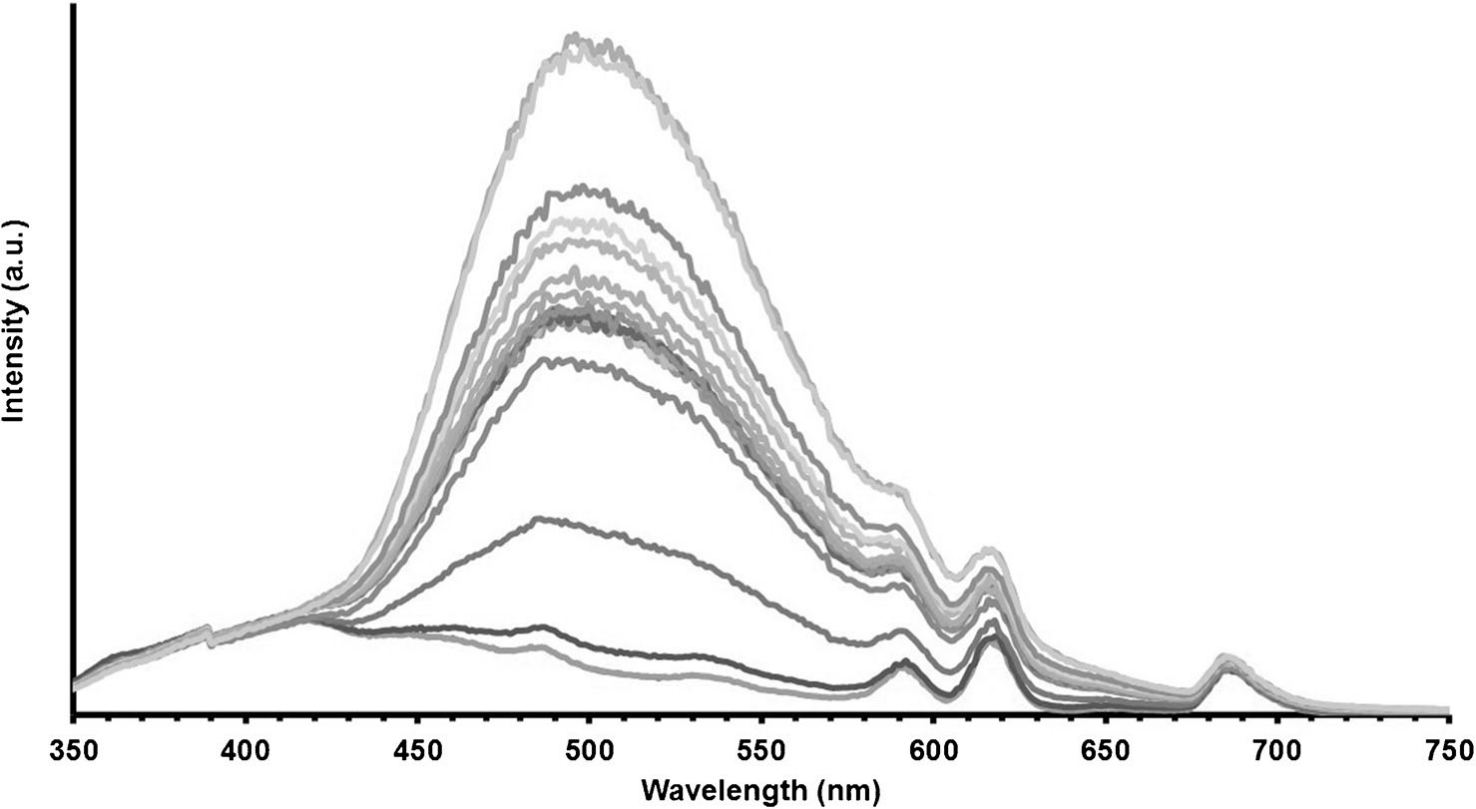
Eu⋅1 versus Zn^II^, fluorescence (1 mM, pH 7.4, 298 K, *λ*=280 nm).

Compound Eu**⋅1** shows a ratiometric fluorescent response to Zn^II^ (Figure 3). Thus, upon ion chelation, there is a large Stokes shift towards the red in emission (by 90 nm from 410 to 500 nm), and a 300 % increase in fluorescence amplitude from the organic fluorophore. These values are thus very similar to those seen in Gd**⋅1** (Figure 2). The inorganic Eu^III^ emission does not change in intensity, when looking at Δ*J*=4 at 685 nm, whereas the other Eu^III^ transitions increase in intensity as a result of being on the tail of the organic AQA Zn^II^-induced emission. It must be noted that there is no increase in energy transfer to the Eu^III^ centre from the AQA chromophore when bound to Zn^II^. This is shown by applying a delay of 0.1 ms and measuring the emission (Figure S5 in the Supporting Information) at which there is no change in intensity. To investigate the mechanism involved in the formation of the emission at 500 nm formed by Zn^II^ chelation, lifetime and quantum yields of the organic fluorophore were measured (Table S2 and Figures S6–S23 in the Supporting Information). It was found that the lifetime for Gd**⋅1** at 410 nm was 2.39 ns and with Zn at 410 nm was 2.32 ns. The lifetime when monitoring at 500 nm increased to 8.82 ns, which is similar to Eu**⋅1**. These data correspond to a significant change in the quantum yield, for example, 0.0018 % for Gd**⋅1** and 0.236 % for Gd**⋅1** Zn (Table S2 in the Supporting Information), which suggests speciation when the zinc is bound. Although these photoluminescent quantum yields (PLQY) are low, it was also found that excitation at 371 nm of either complex in the presence of Zn^II^ yields emission of a single non-lanthanide fluorescence band near 500 nm. Absorbance at a 371 nm wavelength is small, though it is far along the red tail of the absorbance band. Absorption events at the extreme edges of heavily overlapping absorbance bands might arise in majority owing to a single species and are evidenced by single-exponential fluorescence decays that arise owing to selective excitation. Taking together the excitation wavelength dependence of the spectral form of the emission when Zn^II^ is present and the change to the spectral form of the excitation spectrum upon addition of Zn^II^, we suggest that this is indicative of speciation.

**Figure 4:**
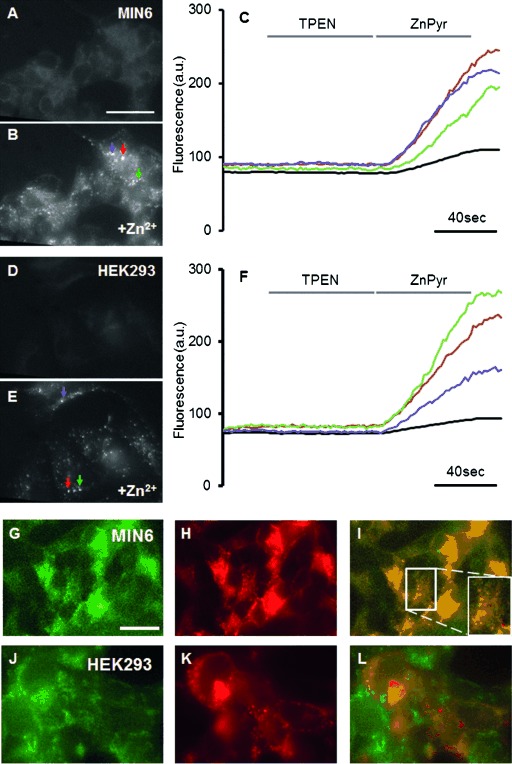
Gd⋅1 localises to intracellular structures in single cells and displays Zn^2+^-dependent changes in fluorescence. Representative images of MIN6 cells stained with A) 50 μM Gd⋅1, incubated in KREBS buffer and B) after 3 min exposure to KREBS with 0.1 mM ZnCl_2_ and 5 μM pyrithione. C) Graph showing the changes in fluorescence of MIN6 cells shown in (A) and (B). Cells were incubated in KREBS buffer with no further additives (frames 20–36), KREBS+50 μM TPEN (TPEN), KREBS+5 μM pyrithione+0.1 mM ZnCl_2_ (ZnPyr) and then KREBS. Black: Whole-cell cluster fluorescence (red, green and purple): fluorescence of selected granules (shown in B). Representative images of HEK293 cells stained with D) 50 μM Gd⋅1 incubated in KREBS buffer and E) 3 min after the addition of 0.1 mM ZnCl_2_ and 5 μM pyrithione. F) Graph showing the changes in fluorescence ratio of HEK293 cells shown in (D) and (E). Cells were incubated in KREBS buffer with no further additives (frames 20–36), KREBS+50 μM TPEN (TPEN), KREBS+5 μM pyrithione+0.1 mM ZnCl_2_ (ZnPyr) and KREBS again. Black: Whole-cell cluster fluorescence (red, green and purple): fluorescence of selected granules (shown in E). G–L) Representative images of MIN6 and HEK293 cells stained with 50 μM Gd⋅1 and infected with an adenovirus encoding NPY-RFP to label dense core secretory vesicles. MIN6 and HEK293 cells were washed twice with KREBS buffer, incubated for 5 min with KREBS+0.1 mM ZnCl_2_ and then imaged. MIN6 cells show staining with G) Gd⋅1, H) NPY-RFP, and I) the two signals are compared with Gd⋅1 in green and NPY-RFP in red with a magnified selection inset. HEK293 cells show staining with J) Gd⋅1, K) NPY-RFP, and L) the two signals are compared with Gd⋅1 in green and NPY-RFP in red. Scale bar: 50 μm.

This ratiometric response of the AQA unit to zinc, combined with no response of the Eu^III^ emission, thus provides a dual fluorescent probe for determining Zn^II^ concentration in cells using the one excitation wavelength on two different timescales, that is, the nano- and milli-second scales. The change in the lanthanide metal centre in the DOTA-based chelate gives the option of a dual-modal zinc sensor for MR/fluorescence with Gd^III^ and bimodal fluorescence with Eu^III^. To determine whether Gd**⋅1** is likely to be tropic towards zinc-containing cells in the pancreas, we first compared uptake into MIN6 (mouse β-cell-derived) cells[29] and HEK293 (non-neuroendocrine) cells. Imaging was performed 30 min after the addition of Gd**⋅1** (Figure 4A, D). Consistent with the high Zn^II^ content of β-cell secretory granules,[21] MIN6 cells show a stronger basal signal than HEK293 cells. Addition of extracellular ZnCl_2_ significantly increased Gd**⋅1** fluorescence in both MIN6 and HEK293 cells (Figure 4B, E), with the strongest fluorescence apparent in punctate structures.

Quantification of this change in fluorescence is shown in Figure 4C and F. Average fluorescence is charted for three bright ‘spots’ chosen from each cell (red, green and purple from Figure 4B and E) as well as the average fluorescence of the cell cluster (black). Fluorescence is not detectably reduced by the addition of the zinc chelator tetrakis(2-pyridylmethyl)ethylenediamine (TPEN), but the addition of zinc and the zinc ionophore pyrithione greatly increased fluorescence. Overall, cell fluorescence was observed to be lower in HEK293 than in MIN6 cells, which is consistent with the enhancement by granular zinc of probe fluorescence in the latter. Interestingly, the low level of fluorescence prior to the addition of zinc/pyrithione indicates a free zinc concentration in this compartment of <1 μM assuming a *K*_d_ for zinc of 22 μM, which is in line with our estimates using protein-based probes.[30]

To ascertain that the bright spots of fluorescence corresponded to the secretory granules, MIN6 and HEK293 cells were infected with an adenovirus encoding neuropeptide Y-red fluorescent protein (NPY-RFP).[31] Gd**⋅1** fluorescence was co-localised with that of NPY-RFP (Figure 4G–L), which is consistent with the accumulation of the zinc probe into secretory granules in β cells. In contrast, there is negligible co-localisation in HEK293 cells (Figure 4J–L), which do not possess dense core secretory granules. The above cellular studies thus show that Gd**⋅1** is cell-permeable and localises in the granules where insulin is stored. Cell-toxicity studies were undertaken to show the biocompatibility of Gd**⋅1**. MIN6 and HEK 293 cells were incubated with and without Gd**⋅1** and show 99.4 and 91.9 % survival, respectively. This study (Figures S27–28 in the Supporting Information), shows that Gd**⋅1** is non-toxic and stable under biological conditions.

We next explored the MR activity of Gd**⋅1**. Phantoms of varying concentrations were prepared and run at 4.7 T (200 MHz; Figure S33 in the Supporting Information) with signal intensity increasing with probe concentration. MRI contrast agents with AQA moieties have previously been shown to bind human serum albumin (HSA).[25] Since this interaction is likely to affect the half-life of Gd**⋅1** in the blood, we determined that this probe binds HSA as predicted. Figure S33B in the Supporting Information shows relaxivity of Gd**⋅1** versus [HSA]; the increase in *r*_1_ shows clear binding of Gd**⋅1** to HSA. The observed increase is due to the larger overall mass of the object and longer *τ*_r_ (rotational correlation time) when Gd**⋅1** binds to HSA. In vivo this will extend the blood half-life of the contrast agent. This has also been observed by Lubag and colleagues, who report on an MR agent that is sensitive to Zn^II^.[13] This is in contrast to our approach developed here, which does not rely on changes in relaxivity dependent upon the secretion of stored zinc from β cells, but rather on the accumulation of the probe into cells. As such, our strategy should provide a means of assessing β-cell mass more than functional β cells. To test this, human islet cells were incubated for 30 min with Gd**⋅1**, and *T*_1_ was measured using a 4.7 T MRI machine. Tubes without cells, which contained only water, gave a *T*_1_ of 2806 ms, tubes with islet cells gave a *T*_1_ of 1156 ms and cells incubated with Gd**⋅1** gave a *T*_1_ of 651 ms. This significant reduction in *T*_1_ suggests that Gd**⋅1** accumulates inside the islet cells and is retained, thus demonstrating that Gd**⋅1** could be a means to assess β-cell mass (Figure S29 in the Supporting Information).

To determine whether Gd**⋅1** might also be accumulated into β cells in vivo, and thus would be useful as a contrast agent (CA) for β-cell mass, in vivo studies were undertaken. Firstly, we performed biodistribution studies in which C57/BL6 mice (*n*=10) were injected with 200 μg of Gd**⋅1**. The organs of the animals were then harvested at two time points: 30 and 180 min after injection (Figure S30 in the Supporting Information). We observed uptake of the agent into the pancreas at 30 min, along with uptake into the kidneys and liver. This biodistribution can be attributed to the small size and neutral charge of the CA. The uptake into the liver presumably reflects binding to HSA (Figure S33b in the Supporting Information) and consequent movement to this organ. Of note, by 180 min these distributions had changed such that there was now a higher concentration of the agent in the pancreas than in the liver. This latter phenomenon might be due to the binding of HSA, thus giving a longer blood half-life, therefore allowing greater accumulation of the agent in the pancreas and other organs. Although the uptake in the pancreas increased over a period of 180 min, the uptake was still low. This is consistent with the probe being selectively taken up by β cells in the islet, which account for only 1–2 % of the pancreas.

**Figure 5:**
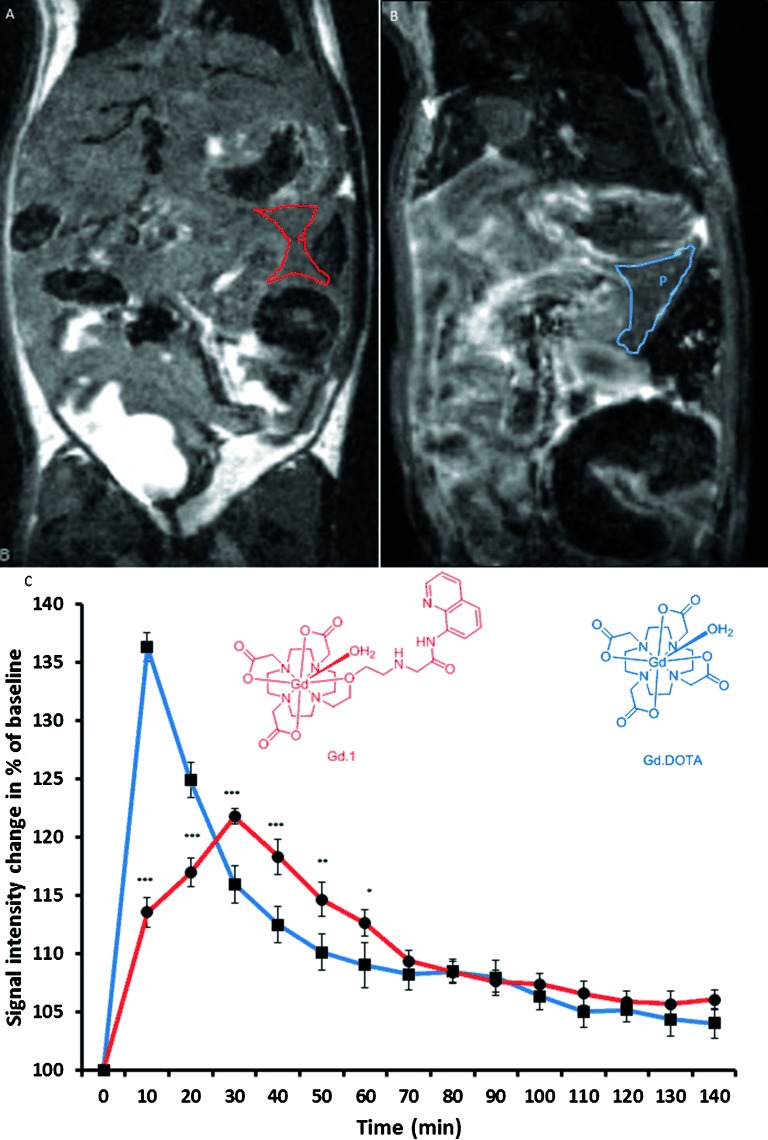
A) MRI image of abdomen of a mouse 40 min after injection with Gd⋅1 (0.1 mmol kg^−1^, pancreas is outlined in red). B) MRI image of abdomen of a mouse 40 min after injection with Gd⋅1 (0.1 mmol kg^−1^, pancreas is outlined in blue). C) Time-course plot of relative signal intensity change in pancreas after administration of Gd⋅1 (red) or Gd⋅DOTA (blue) (0.1 mmol kg^−1^). Structures of Gd⋅1 (red) or Gd⋅DOTA (blue) (*n*=10).

To study further the behaviour of Gd**⋅1** in the living animal, we next undertook in vivo MRI experiments on healthy C57/BL6 mice. Mice were injected with CA (Gd**⋅1** or Gd**⋅DOTA**) at 0.1 mmol kg^−1^ and scanned over 140 min. This revealed an increase in signal intensity of the pancreas over this period (Figure 5). The impact on MRI signals of Gd**⋅1** injection was next compared with that of Gd**⋅**DOTA, the ‘gold standard’, but non-targeted, MRI contrast agent.[22] With Gd**⋅**DOTA a large increase in signal intensity over the pancreatic area was observed at 10 min, then a fast decay was seen as the agent was cleared from blood plasma (Figure 5 and Figure S31 in the Supporting Information). In contrast, at 140 min there was very little increase in signal intensity relative to background. However, changes in the Gd**⋅1** signal were compatible with a gradual uptake of the agent into the pancreas over 30 min, likely reflecting localisation to the β cell and binding to free Zn in secretory granules. This enhancement of the Gd**⋅1** signal relative to the Gd**⋅**DOTA signal continued for approximately 70 min before both signals gradually declined over a time course of 140 min, ultimately resulting in an overall 7 % increase in signal intensity relative to the baseline intensity (Figure 5 and Figure S32 in the Supporting Information).

## Conclusion

We describe a dual-modal imaging agent that senses Zn^II^ with excellent fluorescent and MRI properties, which might potentially be used to monitor β-cell mass in vivo. The dual-modal or dual-fluorescent Zn^II^ probes Gd**⋅1** and Eu**⋅1** show ratiometric fluorescence changes with a large Stokes shift from *λ*_em_=410 to 500 nm upon zinc chelation, and a *K*_d_=22 μM (pH 7.4). Compound Gd**⋅1** has an *r*_1_=4.2 mM^−1^ s^−1^ (9.4 T), which increased up to 6.6 mM^−1^ s^−1^ (9.4 T) upon Zn^II^ binding and down to 4.9 mM^−1^ s^−1^ with a large excess amount of Zn^II^. Examined in β-cell lines, both agents also showed localisation to secretory granules identified by co-expression of granule-resident protein phogrin, and relatively poor uptake into non-β cells, thus reflecting excellent cell permeability, neutral charge and affinity for Zn^II^. The Gd**⋅1** probe reveals an intragranular free Zn^II^ concentration of ≤1 μM, which is consistent with our earlier measurements using non-optimal recombinant protein-based reporters (estimated range for intragranular Zn^II^ of 1–100 μM),[30] thus suggesting that the vast majority of total Zn^II^ within granules (≈30 mM)[20] is complexed to insulin or other species. Although further validation is required, our in vivo studies strongly suggest that Gd**⋅1** is also taken up into the endocrine pancreas, thus making it a promising imaging agent for assessing β-cell mass. We note that previous studies using zinc chelators in vivo in mammals have demonstrated acceptable safety profiles.[32] We therefore believe that Gd**⋅1** provides a first-stage model for the preparation of probes, which might ultimately be of clinical utility.

## Experimental Section

### General procedures

NMR spectra were recorded using a Bruker Avance DRX 400 spectrometer. Chemical shifts are reported in ppm with solvent as internal reference. Electronic absorption spectra were recorded using a PerkinElmer UV/Vis spectrometer.

### Materials

Solvents and starting materials were obtained from Aldrich, Fluka, Acros and Alfa. They were used without further purification unless otherwise stated. Water and H_2_O refer to high-purity water with a resistivity value of 18 MΩ cm^−1^ obtained from the Millipore/MilliQ purification system. Lanthanide chloride salts were purchased from Aldrich. The precise metal ion content was determined by colorimetric titration in acetate buffer (pH 4.5) using a standardised H_2_Na_2_EDTA (EDTA=ethylenediaminetetraacetic acid) solution (Merck) and Xylenol Orange as indicator.

### 2-Chloro-*N*-(quinol-8-yl)acetamide (1)

2-Chloroacetyl chloride (1.3 mL, 16.6 mmol) dissolved in chloroform (30 mL) was added dropwise to a cooled solution of 8-aminoquinoline (2.00 g, 13.9 mmol), pyridine (1.6 mL, 19.4 mmol) and chloroform (90 mL) over a period of 1 h. After stirring for 2 h at room temperature, the mixture was concentrated under reduced pressure and the resultant red-brown solid purified by silica gel column chromatography using dichloromethane to give the product as an off-white solid (2.02 g, 79 %). ^1^H NMR (CDCl_3_): *δ*=10.94 (br, 1 H; NH), 8.89 (dd, ^3^*J*(H,H)=4.2, 4.2 Hz, 1 H; NHCCHCHCH), 8.78 (dd, ^3^*J*(H,H)=4.2, 4.2 Hz, 1 H; NCHCHCH), 8.21 (dd, ^3^*J*(H,H)=8.0, 4.2 Hz, 1 H; NHCCHCHCH), 7.59 (m, 2 H; NHCCHCHCH, NCHCHCH), 7.51 (q, ^3^*J*(H,H)=4.2 Hz, 1 H; NCHCHCH), 4.34 ppm (s, 2 H; CH_2_); ^13^C NMR (CDCl_3_): *δ*=164.5 (CO), 148.5 (NHCCHCHCH), 136.6 (NCHCHCH), 133.5 (NHCCCCH), 128.0 (NHCCHCHCH), 127.3 (NCHCHCH), 122.6 (NHCCHCHCH), 121.8 (NCHCHCH), 117.0 (NHCCHCHCH), 43.4 ppm (CH_2_Cl); IR: 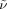
=3325 (NH stretch), 1669 (C—O), 1592 (NH bend), 1328 (C—N), 827 cm^−1^ (C—Cl); LC/MS: *m*/*z*: 221 [*M*+H]^+^.

### 2-(2-Aminoethoxy)ethanol-*N*-(quinol-8-yl)acetamide (2)

Compound **1** (0.24 g, 1.09 mmol), 2-(2-aminoethoxy)ethanol (1.14 g, 10.9 mmol), *N*,*N*-diisopropylethylamine (1.41 g, 10.9 mmol) and a catalytic amount of potassium iodide (0.14 mmol) were heated together under reflux conditions in acetonitrile (70 mL). After 10 h, the mixture was cooled to room temperature and concentrated under reduced pressure to give a yellow oil. This was purified by means of silica gel column chromatography using 20 % MeOH in CHCl_3_ to give the title compound (0.30 g, 90 %). ^1^H NMR (CDCl_3_): *δ*=8.83 (dd, ^3^*J*(H,H)=4.1, 1.6 Hz, 1 H; NHCC*H*CHCH), 8.79 (dd, ^3^*J*(H,H)=7.1, 1.6 Hz, 1 H; NC*H*CHCH), 8.12 (dd, ^3^*J*(H,H)=8.3, 1.4 Hz, 1 H; NHCCHC*H*CH), 7.50 (m, 2 H; NHCCHCHC*H*, NCHCHC*H*), 7.24 (m, 1 H; NCHC*H*CH), 3.72 (m, 4 H; HNC*H*_2_C*H*_2_O), 3.57 (m, 4 H; OC*H*_2_C*H*_2_OH), 2.94 ppm (t, ^3^*J*(H,H)=4.9 Hz, 2 H; C*H*_2_NH); ^13^C NMR (CDCl_3_): *δ*=170.8 (*C*O), 148.5 (NH*C*CHCHCH), 139.0 (NHC*C*N), 136.3 (N*C*HCHCH), 134.2 (NHCC*C*CH), 128.1 (NHCCH*C*HCH), 127.3 (NCH*C*HCH), 121.8 (NHCCHCH*C*H), 121.5 (NCHCH*C*H), 116.7 (NHC*C*HCHCH), 72.3 (CO*C*H_2_NH), 70.7 (OCH_2_*C*H_2_OH), 61.8 (O*C*H_2_CH_2_OH), 53.8 (NHCH_2_*C*H_2_O), 49.4 ppm (NH*C*H_2_CH_2_O); IR: 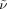
=3285 (OH and NH stretch), 1660 (C—O), 1596 (NH bend), 1324 (C—N), 1118 (C—O), 1062 cm^−1^ (C−O); MS (ESI^+^): *m*/*z*: 290 [*M*+H]^+^.

### 2,2-(Boc)-aminoethoxy-ethanol-*N*-(quinol-8-yl)acetamide (Boc=*tert*-butyloxycarbonyl) (3)

Compound **2** (590 mg, 2.04 mmol), Boc_2_O (534 mg, 2.45 mmol) and Et_3_N (0.28 mL, 2.04 mmol) were stirred in CH_2_Cl_2_ (60 mL) for 12 h. The reaction was monitored by means of TLC and concentrated under reduced pressure and purified by means of column chromatography (CH_2_Cl_2_–MeOH) as a yellow solid (850 mg, 90 %). ^1^H NMR (CDCl_3_): *δ*=10.37 (br, 1 H; N*H*), 8.70 (br, 2 H; NHCC*H*CHCH, NC*H*CHCH), 8.20 (d, ^3^*J*(H,H)=7.7 Hz, 1 H; NHCCHC*H*CH), 7.57–7.48 (m, 3 H; NCHC*H*CH, NHCCHCHC*H*, NCHCHC*H*), 4.23 (m, 2 H), 3.77–3.46 (m, 8 H; NC*H*_2_C*H*_2_O, OC*H*_2_C*H*_2_OH), 1.45 ppm (s, 9 H; (C*H*_3_)_3_); ^13^C NMR (CDCl_3_): *δ*=169.0 (*C*O), 155.3 (*C*O), 148.4 (NH*C*CHCHCH), 138.5 (NHC*C*N), 136.3 (N*C*HCHCH), 134.1 (NHCC*C*CH), 128.0 (NHCCH*C*HCH), 127.4 (NCH*C*HCH), 121.9 (NHCCHCH*C*H), 121.7 (NCHCH*C*H), 116.6 (NHC*C*HCHCH), 81.3 (O*C*(CH_3_)_3_), 72.5 (CO*C*H_2_NH), 70.3 (OCH_2_*C*H_2_OH), 61.7 (O*C*H_2_CH_2_OH), 54.0 (NHCH_2_*C*H_2_O), 48.5 (NH*C*H_2_CH_2_O), 28.2 ppm ((*C*H_3_)_3_); IR: 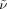
=3326 (OH and NH stretch), 1682 (C—O), 1457 (CH_3_), 1392 (CH_3_), 1246 (C—O), 1165 (C—O), 1136 cm^−1^ (C—O); MS (ESI+): *m*/*z*: 390 [*M*+H]^+^, 412 [*M*+Na]^+^.

### 2,2-(Boc)-aminoethoxy-ethyl-methanesulfonate-*N*-(quinol-8-yl)acetamide (4)

Compound **3** (395 mg, 1.01 mmol) was dissolved in CH_2_Cl_2_ (50 mL) and cooled to 0 °C. Triethylamine (0.42 mL, 3.04 mmol) and methanesulfonyl chloride (0.20 mL, 2.53 mmol) were then added dropwise over 20 min. Following the complete addition of the reagents, the reaction mixture was stirred for a further 10 min and then at room temperature for 1 h. The reaction was monitored by means of TLC, and purified by means of column chromatography (CH_2_Cl_2_–MeOH), 450 mg, 90 %. ^1^H NMR (CDCl_3_): *δ*=10.36 (br, 1 H; N*H*), 8.70 (br, 2 H; NHCC*H*CHCH, NC*H*CHCH), 8.20 (d, ^3^*J*(H,H)=7.7 Hz, 1 H; NHCCHC*H*CH), 7.57–7.48 (m, 3 H; NCHC*H*CH, NHCCHCHC*H*, NCHCHC*H*), 4.15–4.27 (m, 4 H), 3.76–3.60 (m, 6 H), 2.97 (s, 3 H; SO_2_C*H*_3_), 1.45 ppm (s, 9 H; (C*H*_3_)_3_); ^13^C NMR (CDCl_3_): *δ*=168.5 (*C*O), 155.2 (*C*O), 148.4 (NH*C*CHCHCH), 136.3 (N*C*HCHCH), 134.1 (NHCC*C*CH), 128.00 (NHCCH*C*HCH), 127.4 (NCH*C*HCH), 121.8 (NHCCHCH*C*H), 116.4 (NHC*C*HCHCH), 81.3 (O*C*(CH_3_)_3_), 70.4 (OCH_2_*C*H_2_OH), 68.7 (O*C*H_2_CH_2_OH), 54.6 (NHCH_2_*C*H_2_O), 48.6 (NH*C*H_2_CH_2_O), 37.4 (SO_2_*C*H_3_), 28.2 ppm ((*C*H_3_)_3_); IR: 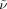
=3326 (NH stretch), 1682 (C—O), 1457 (CH_3_), 1392 (—SO_2_—), 1246 (C—O), 1165 (C—O), 1136 cm^−1^ (C—O); MS (ESI+): *m*/*z*: 490 [*M*+H]^+^, 468 [*M*+Na]^+^.

### *tert-*Butyl 2,2′,2′′-{[2,2-(Boc)-aminoethoxyethyl-*N*-(quinol-8-yl)acetamide]-1,4,7,10- tetraazacyclododecane-1,4,7-triyl} (5)

Compound **4** (71.7 mg, 0.153 mmol) was dissolved in dry acetonitrile (15 mL) and added dropwise to a solution of triester cyclen (94.7 mg, 0.184 mmol) and triethylamine (0.11 mL, 0.767 mmol) in dry acetonitrile (35 mL). After heating under reflux conditions for 72 h, the resulting reaction mixture was concentrated under reduced pressure to give a thick brown oil, which was purified by means of column chromatography using silica gel (CH_2_Cl_2_/CH_3_OH 95:5) to yield the title compound as a viscous light brown oil (0.104 g, 77 %). ^1^H NMR (CDCl_3_): *δ*=10.29 (br, 1 H; NC*H*CHCH), 10.06 (br, 1 H; *H*N—CO), 8.78 (m, 2 H; NHCCHC*H*CH,), 8.21 (m, 1 H; NCHC*H*CH), 7.56–7.50 (m, 2 H; NHCCHCHCH, NCHCHCH), 4.13 (2 H; C*H_2_*CO), 3.65–3.60 (m, 4 H; C*H_2_*NCO), 3.45 (br, 2 H; C*H*_2_O), 4.36–3.46 (16 H; 6,8-C*H*_2_, 5,9-*CH*_2,_ 2,12-*CH*_2_, 3,11-*CH*_2_), 1.42 (s, 9 H; C*H*_3_), 1.46 ppm (m, 27 H; C*H*_3_); ^13^C NMR (CDCl_3_): *δ*=172.6 (*C*O), 170.5 (*C*O), 169.7 (*C*O), 168.1 (*C*O), 148.4 (NH*C*CHCHCH), 138.3 (NHC*C*N), 136.5 (N*C*HCHCH), 128.0 (NHCCH*C*HCH), 127.3 (NCH*C*HCH), 121.9 (NHCCHCH*C*H), 116.3 (NHC*C*HCHCH), 82.3 (O*C*(CH_3_)_3_), 81.8 (*C*((CH_3_)_3_)_2_), 81.6 (*C*(CH_3_)_3_), 76.1 (CO*C*H_2_NH), 70.0 (OCH_2_*C*H_2_OH), 58.2 (*C*O(CH_3_)_3_), 56.5 (O*C*H_2_CH_2_OH), 55.7 (NHCH_2_*C*H_2_O), 52.7 (NH*C*H_2_CH_2_O), 51.3 (*C*O(CH_3_)_3_), 50.3 (6,8-*C*H_2_), 49.2 (5,9-*C*H_2_), 48.4 (2,12-*C*H_2_), 47.5 (3,11-*C*H_2_), 28.2 (*C*H_3_), 28.0 (*C*H_3_)_2_, 27.9 ppm ((*C*H_3_)_3_); ESI-MS: *m*/*z*: 887 [*M*+H]^+^, 909 [*M*+Na]^+^.

### 2,2′,2′′-{[2-Aminoethoxyethyl-*N*-(quinol-8-yl)acetamide]-1,4,7,10-tetraazacyclododecane-1,4,7- triacetic acid} (6)

Compound **5** (50 mg, 0.0564 mmol) was dissolved in dichloromethane (3 mL) and trifluoroacetic acid (3 mL) was added dropwise. After stirring overnight at room temperature, the volatile compounds were removed under a stream of N_2_. The oily residue was dissolved in water and washed with dichloromethane. The aqueous layer was reduced under vacuum to leave a light yellow oil. ^1^H NMR (D_2_O): *δ*=8.95 (d, ^3^*J*(H,H)=5.9 Hz, 1 H; NCHCHCH), 8.63 (d, ^3^*J*(H,H)=7.3 Hz, 1 H; NHCCHCHCH), 8.41 (d, ^3^*J*(H,H)=9.8 Hz, 1 H), 7.77 (d, ^3^*J*(H,H)=7.6 Hz, 1 H; NCHCHCH), 7.63 (m, 2 H; NHCCHCHCH, NCHCHCH), 4.36–3.46 ppm (32 H; CH_2_—CO, CH_2_—NCO, 6,8-C*H*_2_, 5,9-*CH*_2,_ 2,12-*CH*_2_, 3,11-*CH*_2_, HNC*H_2_*C*H_2_*O, OC*H_2_*C*H_2_*N); ^13^C NMR (D_2_O): *δ*=174.1 (*C*O), 167.2 (*C*O), 162.8 (*C*O), 148.4 (NHCCH*C*HCH), 144.8 (N*C*HCHCH), 133.6 (NHC*C*N), 133.1 (NH*C*CHCHCH), 130.0 (NCH*C*HCH), 129.9 (NHCCHCH*C*H), 129.2 (*C*HN), 125.9 (C*C*CH) 122.2, (NHC*C*HCHCH), 65.9 (NH CO*C*H_2_), 65.7 (OCH_2_*C*H_2_NH), 54.5 (*C*O), 53.5 (O*C*H_2_CH_2_N), 52.9 (N*C*H_2_CO), 51.6 (N*C*H_2_CO), 48.3 (N*C*H_2_CO), 47.9 (ring-*C*H_2_), 47.1 ppm (ring-*C*H_2_); MS (ESI+): *m*/*z*: 618 [*M*+H]^+^.

### Gd⋅2,2′,2′′-{[2-aminoethoxy-ethyl-*N*-(quinol-8-yl)acetamide]-1,4,7,10-tetraazacyclododecane- 1,4,7-triacetate} (Gd⋅1)

Compound **6** (0.031 g, 0.050 mmol**)** was dissolved in H_2_O (5 mL) at room temperature. Following the addition of GdCl_3_**⋅**6 H_2_O (0.018 g, 0.050 mmol), the pH of the solution was adjusted to 5.5 by NaOH (1 M). After 5 h, the pH was further adjusted to 10.8 and left for 40 min. The white precipitate was centrifuged and the supernatant separated and concentrated under reduced pressure to exactly 1 mL. The complex was purified using a Sephadex G-25 size exclusion column, eluting with water. The aliquots that contained the complex were combined and the desired complex was obtained as a hygroscopic solid (0.039 g 91 %). MS (ESI+): *m*/*z*: 773 [*M*+H]^+^; MS (ESI+): *m*/*z* calcd for C_29_H_41_GdN_7_O_8_: 773.2179; found: 773.2241.

### Eu⋅2,2′,2′′-{[2-aminoethoxy-ethyl-*N*-(quinol-8-yl)acetamide)]-1,4,7,10-tetraazacyclododecane-1,4,7-triacetate} (Eu⋅1)

Compound **6** (0.031 g, 0.050 mmol) was dissolved in H_2_O (5 mL) at room temperature. Following the addition of EuCl_3_**⋅**6 H_2_O (0.017 g, 0.050 mmol), the pH of the solution was adjusted to 5.5 by NaOH (1 M). After 5 h, the pH was further adjusted to 10.8 and left for 40 min. The white precipitate was centrifuged and the supernatant separated and concentrated under reduced pressure to exactly 1 mL, then the complex was purified using a Sephadex G-25 size exclusion column and eluting with water. The aliquots that contained the complex were combined and the desired complexes were obtained as a hygroscopic solid (0.038 g 90 %). ^1^H NMR (400 MHz, D_2_O, 278 K): *δ*=26.45, 21.84, 21.48, 18.59, 17.21, 16.01, 15.08, 13.38, 12.76, 12.06, 8.78, 8.25, 7.95, 7.83, 7.10, 6.26, 6.18, 3.45, 2.83, 1.90, 0.64, 0.35, −0.39, −1.65, −2.65, −3.17, −4.22, −4.47, −6.24, −7.74, −7.96, −9.07, −9.30, −10.44, −11.19, −13.55, −15.43, −16.29, −18.80, −20.24, −22.36 ppm; MS (MALDI-TOF): *m*/*z*: 768 [*M*+H]^+^.

### Relaxivity measurements

Compound Gd**⋅1** was prepared in situ by mixing the appropriate amounts of ligand and GdCl_3_**⋅**6 H_2_O (99.99 %; Aldrich) in H_2_O followed by adjustment of the pH with NaOH aqueous solution (pH 7.4) for relaxivity measurements. The resulting solution was placed in a 1.7 mm diameter capillary, which was sealed. The absence of free gadolinium was checked in all samples by the xylenol orange test. The 1/*T*_1_ measurements were performed using a Bruker DRX 400 spectrometer (400 MHz).

### Luminescence spectroscopy

Luminescence measurements (spectra and lifetimes) were recorded using a Cary Varian eclipse luminescence spectrometer. The excitation source was a 450 W Xe arc lamp and all spectra were corrected for detection and optical spectral response (instrumental functions) of the spectrofluorimeter. Phosphorescence lifetimes were measured in the time-resolved mode. They are averages of three independent measurements that were taken by monitoring the decay at the maxima of the emission spectra. The monoexponential decays were analysed by using Graph Pad Prism 5. Samples were held in a 10×10 nm or 10×4 nm quartz Hellma cuvette and cutoff filters were used to avoid second-order diffraction effects.

### Metal-binding titrations

**Relaxivity**: Compound Gd**⋅1** was prepared as above at 1 mM concentration to which ZnCl_2_ in H_2_O was added to give the appropriate concentration of Zn to Gd**⋅1**. The 1/*T*_1_ was measured as above.

**Fluorescence and UV/Vis spectroscopy**: Compound Gd**⋅1/**Eu**⋅1** was prepared at 0.1 mM concentration to which ZnCl_2_ or CaCl_2_ in 10 mmol HEPES buffer at pH 7.4 were added to give the appropriate concentration of Zn to Gd**⋅1/**Eu**⋅1**. The data were fitted on Graph Pad Prism and using an iterative least-square fitting procedure operating in Microsoft Excel.

**Fluorescence lifetimes**: Recorded using time-correlated single-photon counting (TCSPC) in 10 mm quartz cuvettes. Excitation sources were a Horiba JY NanoLED pulsed diode laser with 1 MHz repetition rate at nominal wavelength of 371 nm, or a Coherent Verdi-pumped, frequency-trebled MIRA-900 Titanium-Sapphire laser with a pulse-switch radiofrequency cavity dumper and nominal wavelength of 296 nm. Fluorescence was detected orthogonally to the excitation path and determined using a Jobin Yvon Triax 190 monochromator. The instrument response was recorded at the excitation wavelength with Ludox colloidal silica as the scattering medium in a 10 mm quartz cuvette. Fluorescence was recorded with a minimum of 10 000 counts at the peak of the pulse height analyser and the data were fitted by iterative reconvolution of a sum of exponential functions with the instrument response. The reduced *χ*^2^ parameter quantifies the goodness-of-fit, the Durbin–Watson parameter quantifies the autocorrelation, and the randomness of residuals and auto-correlated residuals permit a secondary analysis of both the goodness-of-fit and the true state of the autocorrelation function.

**Fluorescence quantum yields**: Measured using the relative method over at least four different concentrations described in detail elsewhere.[33] For the analyte emission near 410 nm, the standard was quinine sulphate in aqueous 0.1 M sulphuric acid (*ϕ*_r_=0.55), and for the emission at 500 nm, the standard was fluorescein in aqueous 0.1 M sodium hydroxide (*ϕ*_r_=0.89). Solutions of LnM were prepared in 10 mM HEPES buffer, with 1 equivalent of zinc sulfate. Absorbance was measured at 312 nm to avoid absorbance by the buffer and all solutions were excited at this wavelength for emission spectra. Emission spectra were fully corrected for instrument response and background fluorescence and integrated between constant limits. Emission near 410 nm was integrated between 360 and 420 nm, and emission near 500 nm was integrated between 500 and 550 nm. All integrated bands had an uncorrected detector response within the linear range of the detector. Linear least-squares regression of integrated emissions versus 1–10^−A^ gave *R*^2^ goodness-of-fit parameters of greater than 0.99. The quantum yields were calculated according to Equation (1) below:

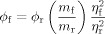
(1)

in which *ϕ*_f,r_ are the quantum yields (f=sample, r=reference), *m*_f,r_ are the gradients of integrated emission versus 1–10^-A^ and *η*_f,r_ are their refractive indices (*η*_f,r_=1.33).

**pH titrations**: A 1 mM solution of Eu**⋅1** in 0.1 M KCl (3 mL) was prepared. The pH of the solution was monitored and adjusted to acidic or basic conditions using aliquots of 1 M HCl or 1 M NaOH solution, respectively. The pH was allowed to stabilise. The fluorimeter was operated using an excitation slit width of 20 nm and an emission slit width of 5 nm. An excitation wavelength of 325 nm was used. The same was also carried out with Eu**⋅1** and 1 equivalent of Zn.

### In vitro cell growth and imaging

HEK293 cells were cultured in Dulbecco’s modified eagle’s medium (DMEM; Lonza) supplemented with 10 % fetal bovine serum (FBS; Lonza), 1 % glutamine (Lonza) and 1 % penicillin/streptomycin (Gibco). MIN6 cells were cultured in DMEM supplemented with 15 % FBS, 1 % glutamine, 1 % penicillin/streptomycin, 50 μM β-mercaptoethanol (Fluka) and 10 mM 4-(2-hydroxyethyl)-1-piperazineethanesulfonic acid (HEPES). Two days prior to imaging, cells were grown in coverslips in six-well flat-bottomed plates. For NPY-RFP studies, cells were infected with 1.5×10^5^ pfu of an adenovirus encoding NPY-RFP the day before visualisation.[31]

On the day of visualisation, cells were washed twice with KREBS buffer (140 mM NaCl, 3.6 mM KCl, 0.5 mM NaH_2_PO_4_, 0.2 mM MgSO_4_, 1.5 mM CaCl_2_, 10 mM HEPES (pH 7.4), 2 mM NaHCO_3_) pre-equilibrated with 95:5 O_2_/CO_2_ that contained 25 mM glucose. They were then incubated with KREBS+50 μM Gd**⋅1** for 30 min and washed twice with KREBS buffer. Cells were visualised every 5 s for 10 min. After 3 min (36 images), 50 μM *N*′,*N*′,*N*′-tetrakis(2-pyridylmethyl)ethylenediamine (TPEN) was added. After 6 min (72 images), TPEN was removed and 0.1 mM ZnCl_2_ and 5 μM pyrithione were added. After 9 min (108 images), ZnCl_2_ and pyrithione were removed. Images were captured using an Olympus IX-70 wide-field microscope with a 40× oil immersion objective and an Imago charge-coupled device camera (Till Photonics, Grafelfing, Germany) controlled by TILLvisION software (Till Photonics) or with an sCMOS camera (Zyla, Andor) controlled by micromanager.[34] For all images, microscope settings such as brightness, contrast and exposure time were held constant to compare the relative intensity of Gd**⋅1** and NPY-RFP.

For viability assays, HEK293 and MIN6 cells were grown overnight as above. Ninety minutes prior to visualisation, cells were either incubated in KREBS buffer or KREBS buffer that contained 50 μM Gd**⋅1** and grown for an hour. Thirty minutes before visualisation, propidium iodide (3 μL) and 1.5 μM Calcein-AM were added to each 2 mL well. Cells were visualised using a Zeiss Axiovert confocal microscope coupled to a Nipkow spinning-disk head (Yokogawa CSU-10) using a 40× air objective. A solid-state laser (CrystaLaser) controlled using a laser-merge module (Spectral Applied Physics) provided wavelengths of 491 nm to excite propidium iodide, and wavelengths of 561 nm to excite Calcein-AM. Images were captured using a highly sensitive 16-bit, 512×512 pixel back-illuminated EM-CCD camera (ImageEM 9100-13; Hamamatsu). Volocity software (PerkinElmer) provided the user interface.

After initial acquisition Triton X-100 (0.2 %) was added to each well and 10 min later cells were visualised to check for effective PI staining and removal of Calcein-AM staining. For all images, microscope settings such as brightness, contrast and exposure time were held constant.

### Magnetic resonance imaging

Compound Gd**⋅1** (1 mL) in Eppendorf tubes at concentrations of 0.05, 0.3, 0.75, 0.1, 0.25, 0.5 and 1 mM were placed in a 4.7 T MRI scanner. A *T*_1_ inversion recovery experiment was undertaken to provide the images seen in Figure S31 of the Supporting Information.

Human serum albumin (HSA) at increasing concentrations was added to Gd**⋅1** (1 mL, 0.1 mM) and were placed in a 4.7 T scanner and a *T*_1_ inversion recovery experiment was undertaken to give the data seen in Figure S31 of the Supporting Information.

Human islets (Diabetes Research Institute OSR-DRI, San Raffaele Scientific Institute) were isolated post mortem from heart-beating donors with suitable ethical permissions and used with local ethical committee approval (NRES Committee London, Fulham 07/H0711/114). Islets were incubated in either 2 mL islet media (RPMI supplemented with 10 % FBS and 1 % penicillin/streptomycin), or 2 mL islet media that contained X = 1 mM Gd**⋅1** for 2 h. The islets were washed twice with 1 mL islet media, transferred to a 50 μL tube, and any remaining media were removed. The islet pellet was sealed in place by covering with 4 % low-melting point agarose in PBS (50 μL). A second control consisted of 4 % low-melting point agarose in PBS (100 μL).

### In vivo studies

In vivo experiments were conducted in coherence to Swiss animal welfare legislation. The experiment was covered by the animal license BS-1418. The study was conducted with Balb/C mice of 19 months of age (BW approx. 20–24 g) (*n*=10 animals).

**Anaesthesia**: Isoflurane 1–3 vol % in O_2_ induced outside the magnet in an anaesthesia box. A tail vein catheter was placed under anaesthesia outside of the magnet and the animal immediately thereafter was placed on a cradle in the magnet. The animal/pancreas was fixed with tape to reduce respiration movement.

### Localisation of pancreas with TriPilot and TriPilot-multi protocols

**TriPilot (12 s 800 ms)**: Gradient echo sequence with TE: 3 ms, TR: 100 ms, FoV: 60×60 mm, slice thickness: 2.00 mm, slices: 1, matrix size: 256×256.

**TriPilot-multi (25 s 600 ms)**: Gradient echo sequence with TE: 3 ms, TR: 200 ms, FoV: 60×60 mm, slice thickness: 1.00 mm, three orthogonal sections with slices: five each, matrix size: 256×256. Two baseline acquisitions with T1wMSME followed by Gd**⋅1** or DOTAREM infusion and a series of 14 consecutive T1wMSME acquisitions every 10 min for 2 h 20 min were performed.

**T1wMSME (8 min 57 s 600 ms)**: Spin-echo sequence with TE: 14.2 ms, TR: 350 ms, FoV: 50×50 mm, slice thickness: 0.6 mm, slices centred on the pancreas: 16, matrix size: 256×192, NA=8. Slow in vivo infusion of Gd**⋅1** at 0.1 mmol kg^−1^ or DOTAREM at 0.1 mmol kg^−1^ was performed in the tail vein by a remote infusion pump with the animal positioned in the magnet.
