# Supplementary material for: Dual-Modal Magnetic Resonance/Fluorescent Zinc Probes for Pancreatic β-Cell Mass Imaging
Source: Chemistry. 2015 Mar 3;21(13):5023–33. doi: 10.1002/chem.201406008 (PMC4464533; doi:10.1002/chem.201406008)
Supplement: Supplementary file 1 [file chem0021-5023-sd1.pdf]

CHEMISTRY
A European Journal

Supporting Information

Dual-Modal Magnetic Resonance/Fluorescent Zinc Probes for Pancreatic β-Cell Mass Imaging

Graeme J. Stasiuk,[a] Florencia Minuzzi,[b] Myra Sae-Heng,[a] Charlotte Rivas,[a] Hans-Paul Juretschke,[c] Lorenzo Piemonti,[d] Peter R. Allegrini,[e] Didier Laurent,[f] Andrew R. Duckworth,[g] Andrew Beeby,[g] Guy A. Rutter,*[b] and Nicholas J. Long*[a]

chem_201406008_sm_miscellaneous_information.pdf

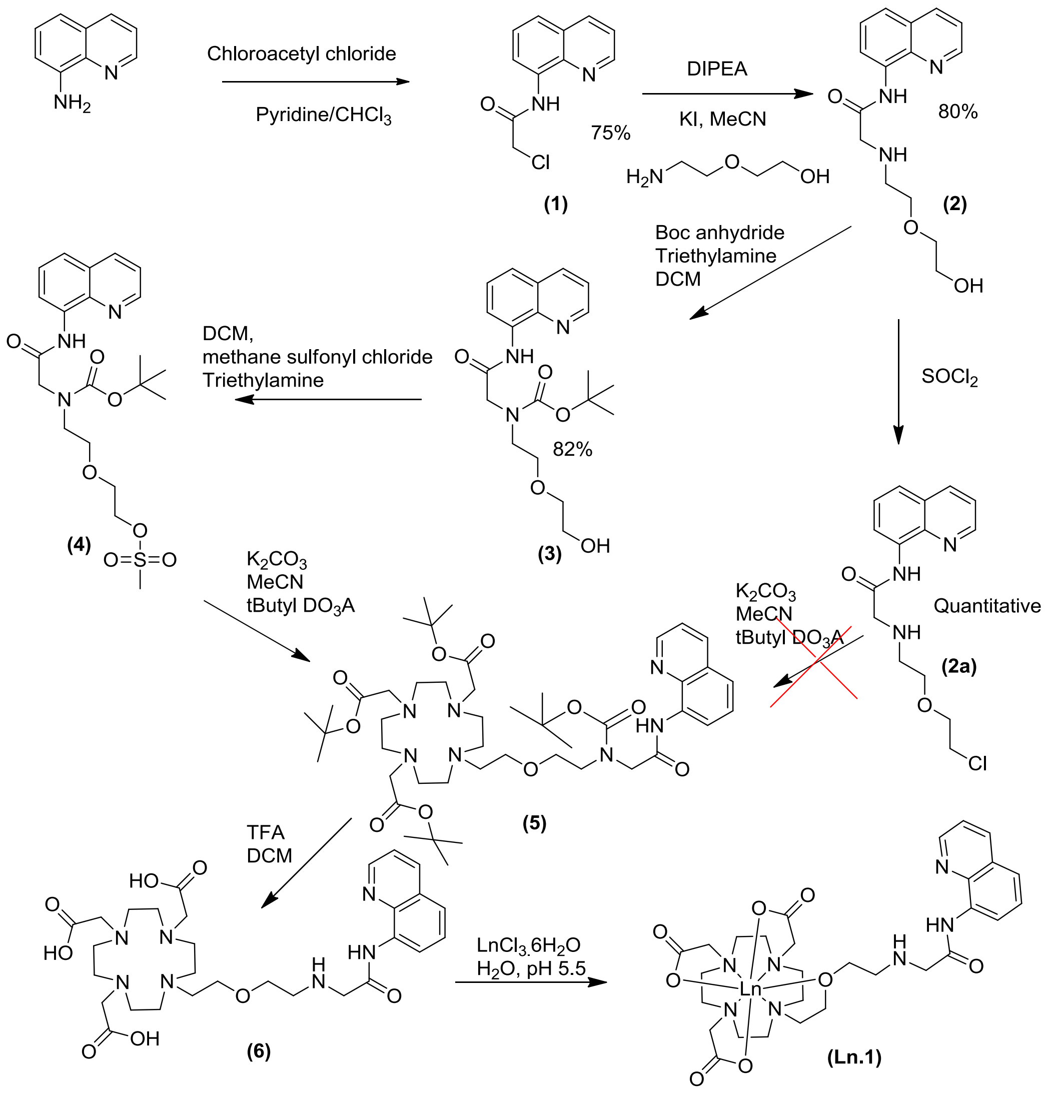

Figure S1 Formation of Gd.1 and Eu.1

Synthesis of 2, 2-Aminoethoxy-chloroethane-N-(quinol-8-yl)acetamide (2a)

2 (0.20 g, 0.69 mmol) was stirred with 1, 2 – dichloroethane (50 ml), and saturated with HCl gas for 30 minutes. The resulting yellow solution was then cooled with an ice bath, and thionyl chloride (0.22 g, 1.87 mmol) added dropwise. The mixture was then warmed to room temperature, and heated to 60 ^{0}C for 1 hour. After which, the reaction mixture was concentrated under reduced pressure to leave an orange-red solid, which was recrystallized using ethanol and ether to give the title compound (0.22 g, 99%). ^{1}H NMR ($CDCl_3$): δ = 9.12 (1 H, dd, $^{3}J_{HH}$ = 4.7 Hz, $^{3}J_{HH}$ = 1.2, NHCC*H*CHCH), 8.90 (1 H, d, $^{3}J_{HH}$ = 8.2 Hz, NC*H*CHCH), 8.41 (1 H, d, $^{3}J_{HH}$ = 7.4, NHCCHCHC*H*CH), 8.08 (1 H, d, $^{3}J_{HH}$ = 8.2 Hz, NHCCHCHCH*C*H), 7.95 (1 H, dd, $^{3}J_{HH}$ = 8.2 Hz, $^{3}J_{HH}$ = 4.8 Hz, NCHCHC*H*), 7.86 (1 H , t, $^{3}J_{HH}$ = 7.9 Hz, NCHC*H*CH), 4.37 (2 H, s, C*H*$_2$NH), 3.88 (4 H, m, HNC*H*$_2$C*H*$_2$O), 3.78 (2 H, t, $^{3}J_{HH}$ = 5.3 Hz, OC*H*$_2$CH$_2$Cl), 3.46 (2 H, t, $^{3}J_{HH}$ = 4.9 Hz, OCH$_2$C*H*$_2$Cl). MS(ESI^{+}): *m/z* = 308 amu [M+H]$^{+}$. IR, v_{max}/cm^{-1}: 3202 (NH stretch), 1737 (C=O).

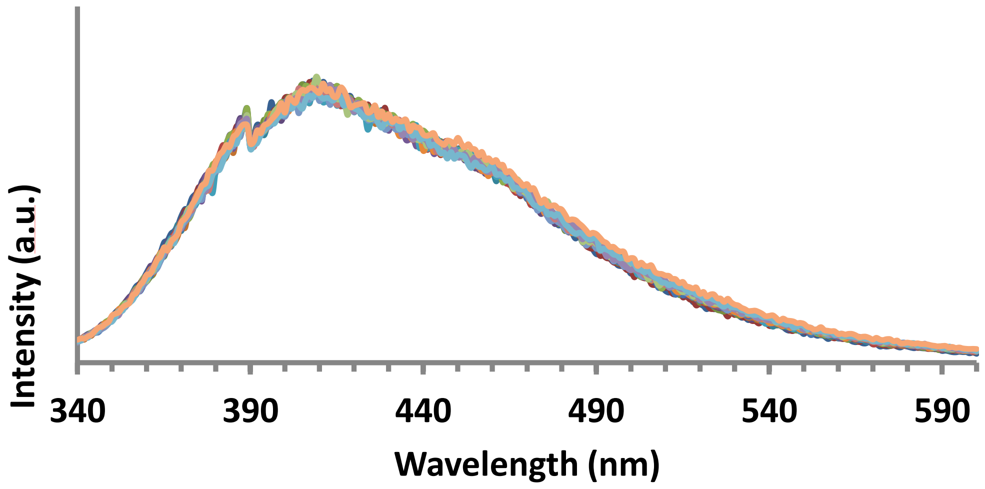

Figure S2. **Gd.1** vs Ca(II) Fluorescence, λ_{ex} 350 nm (pH 7.4, 1 mM **Gd.1**, $CaCl_2$ 0-5 mM, 298 K)

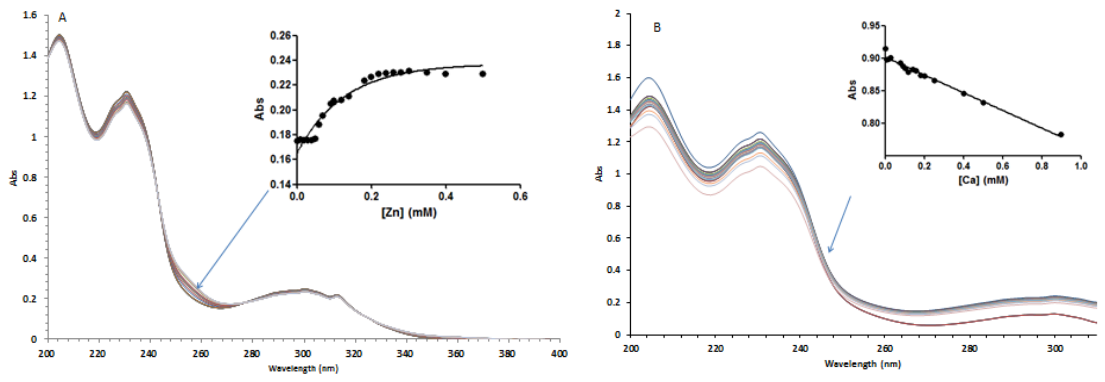

Figure S3 Gd.1 *vs* Zn (A) and vs Ca (B) UV-vis titration, (0.1 mM, pH 7.4, HEPES, 298K)

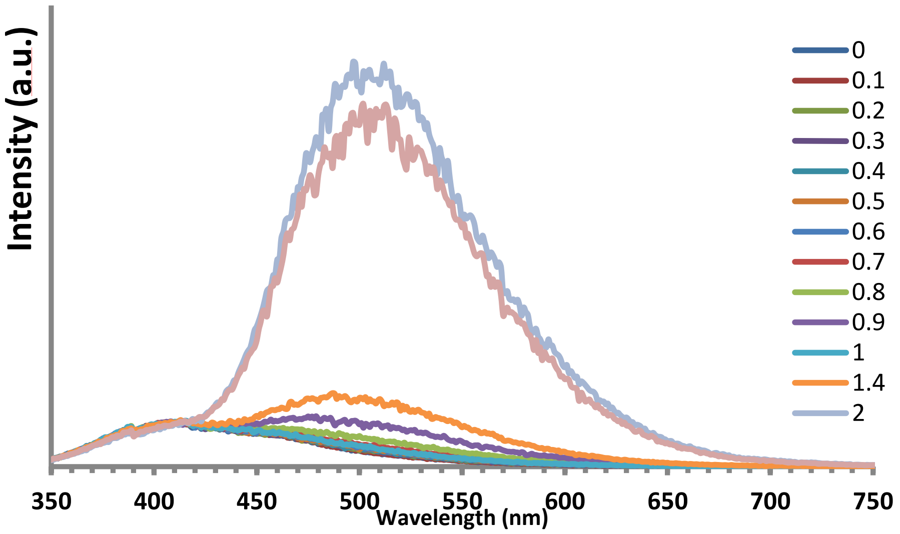

Figure S4 Gd.1. Ca(II) (5 equiv.) *vs* Zn(II) (0 → 2 equiv.) fluorescence titration, λ_{ex} = 350 nm (1 mM, pH 7.4, 298 K)

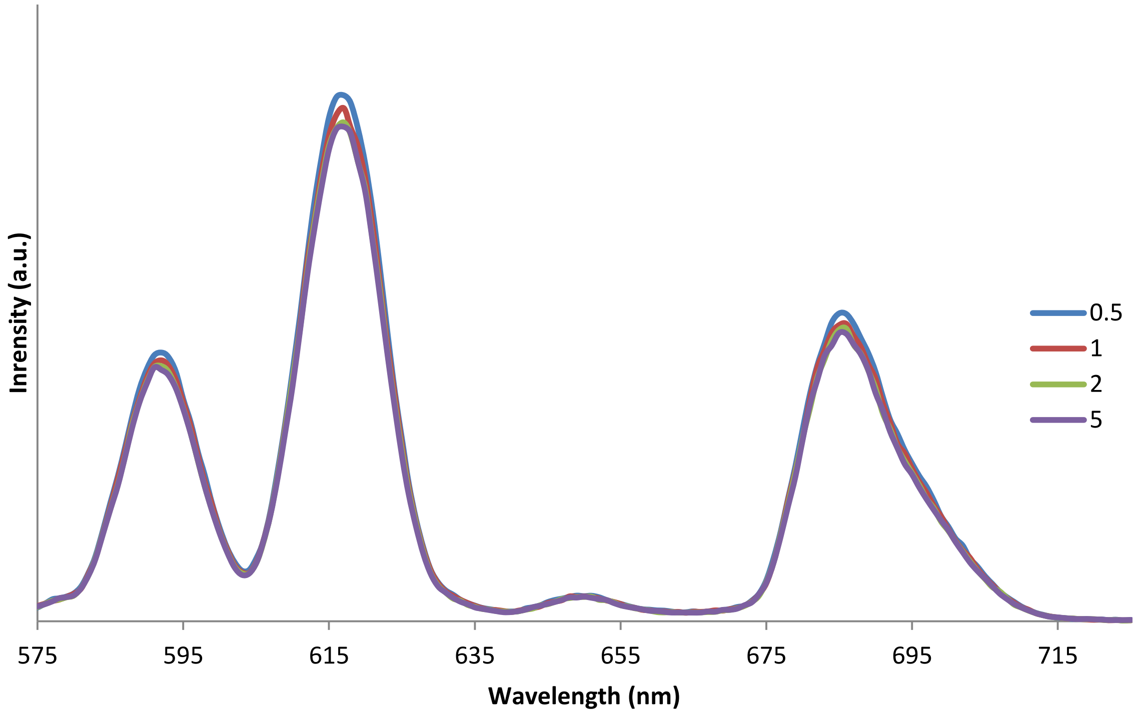

Figure S5 Eu.1 *vs* Zn fluorescence titration, λ_{ex} = 350 nm, 0.1 ms delay (1 mM, pH 7.4, 298 K)

Compound	λ_{ex}	λ_{em}	k_{H2O}	k_{D2O}	q
**Eu.1**	280/252/395	616	1.86846	0.667111	1.14
**Eu.1 Zn(0.5)**	280/252/395	616	1.842299	0.637755	1.14
**Eu.1 Zn(1)**	280/252/395	616	1.831502	0.636537	1.13
**Eu.1 Zn(2)**	280/252/395	616	1.867762	0.646412	1.16
**Eu.1 Zn(5)**	280/252/395	616	1.846381	0.648088	1.14

Table S1. Lifetime data for **Eu.1 plus Zn**.

The lifetime data were fitted using a non-linear regression on graph pad prism. The hydration state was calculated using the equation below:[S2]

$$q = 1.2((k_{H_2O}-k_{D_2O})-0.25)$$

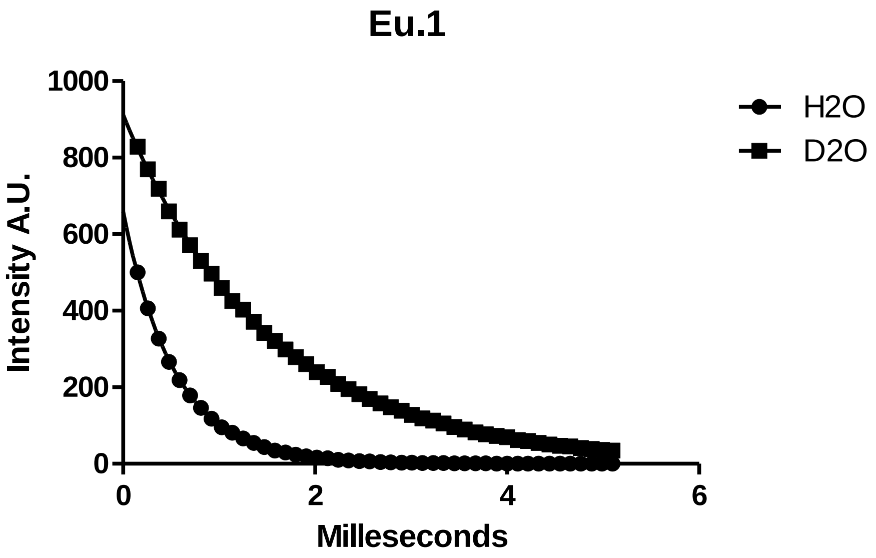

Figure S6 Lifetime measurements of **Eu.1** in H_2O and D_2O (pH 7.2, 298 K)

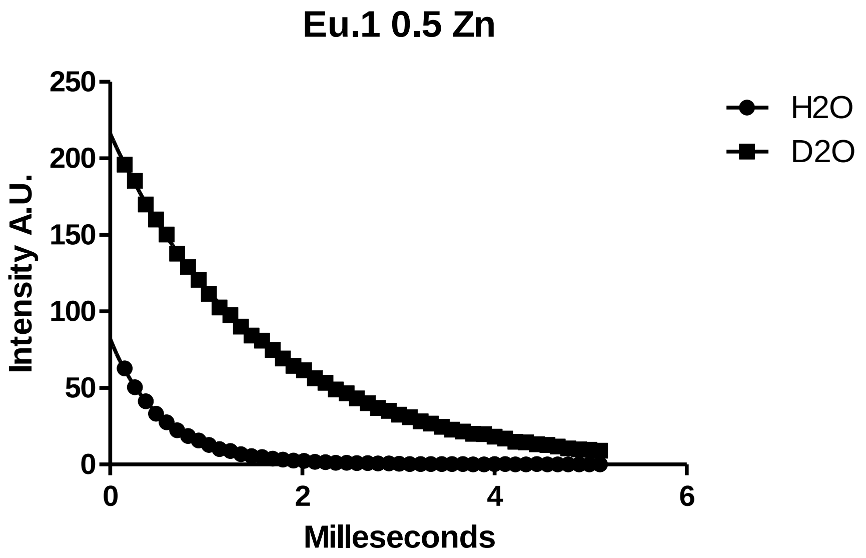

Figure S7 Lifetime measurements of **Eu.1 0.5 Zn** in H_2O and D_2O (pH 7.2, 298 K)

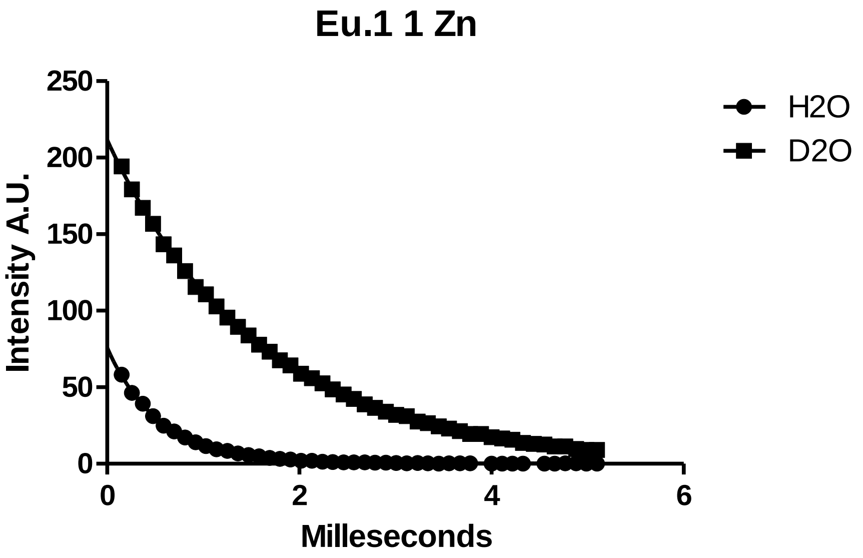

Figure S8 Lifetime measurements of **Eu.1 1 Zn** in H_2O and D_2O (pH 7.2, 298 K)

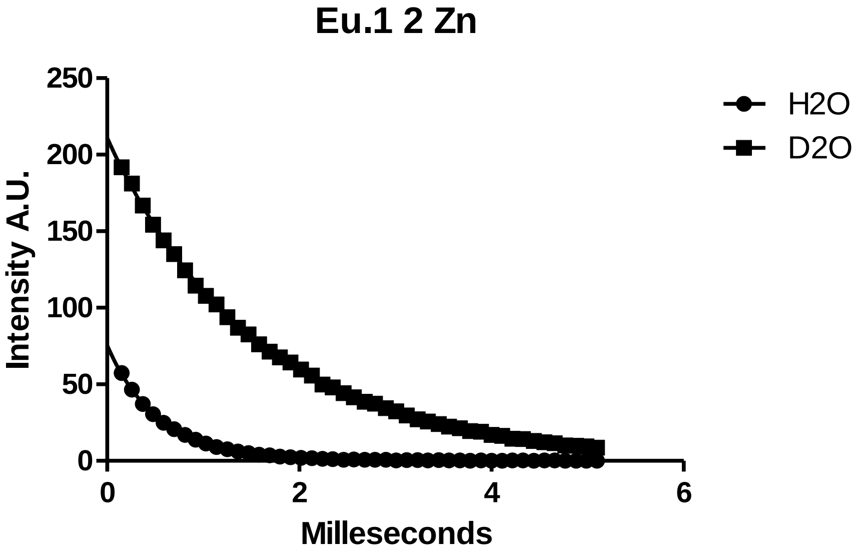

Figure S9 Lifetime measurements of **Eu.1 2 Zn** in H_2O and D_2O (pH 7.2, 298 K)

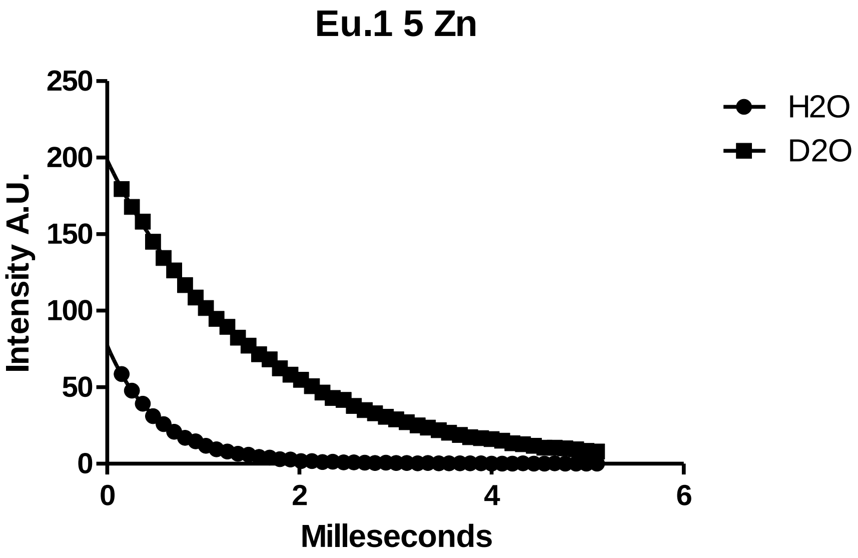

Figure S10 Lifetime measurements of **Eu.1 5 Zn** in H_2O and D_2O (pH 7.2, 298 K)

Species	τ_1 (f_1)[a] /ns	τ_2 (f_2) /ns	χ^2	D.W.	ϕ_f[b]	λ_{ex}/nm	λ_{em}/nm
Eu.1	3.34 (0.72)	0.43 (0.28)	0.95	1.75	0.0068	296	410
Eu.1 + Zn(II)	6.14 (0.77)	0.92 (0.23)	1.06	1.70	0.0069	296	410
Eu.1 + Zn(II)	7.40 (0.84)	1.39 (0.16)	1.02	1.82	0.0219	296	500
Eu.1 + Zn(II)	7.66 (0.95)	1.24 (0.05)	1.16	1.66	-	371	500
Gd.1	2.39 (0.41)	0.32 (0.59)	1.01	2.01	0.0018	296	410
Gd.1 + Zn(II)	2.32 (0.50)	0.35 (0.50)	1.05	2.07	0.0018	296	410
Gd.1 + Zn(II)	7.12 (0.75)	1.58 (0.25)	0.99	1.95	0.2364	296	500
Gd.1 + Zn(II)	8.82 (0.99)	0.88 (0.01)	1.13	1.69	-	371	500

[a]fraction of integrated convolution accounted for by this component [b]excitation at 312 nm

Table S2. Organic fluorescence lifetimes and quantum yields.

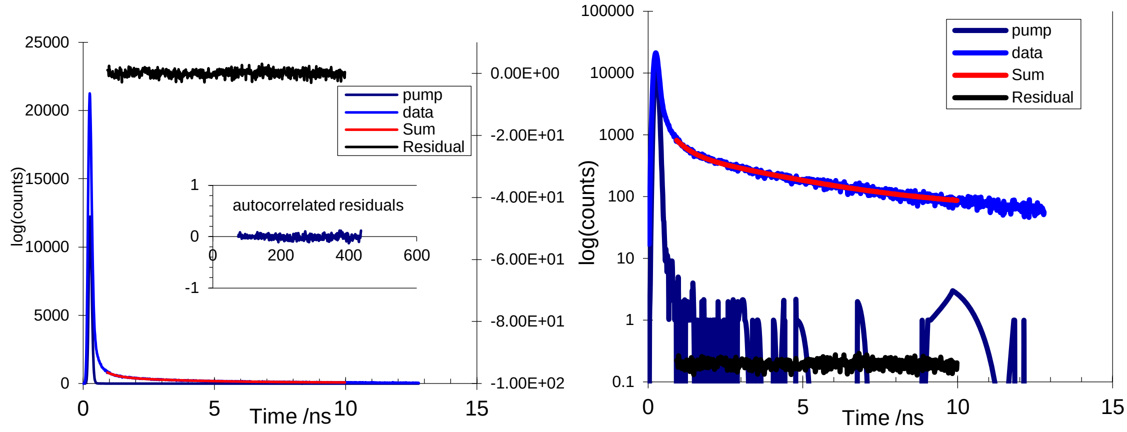

Figure S11 Lifetime decay for **Eu.1** at 410 nm (ex = 296 nm)

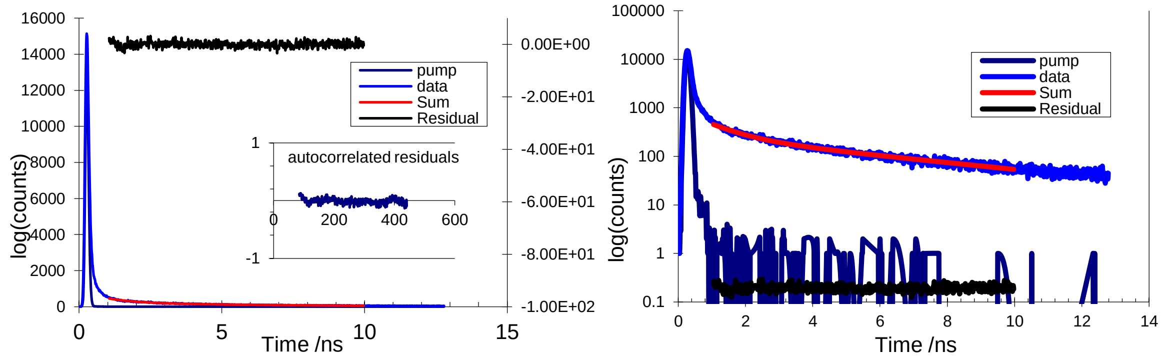

Figure S12 Lifetime decay for **Eu.1 Zn** at 410 nm (ex = 296 nm)

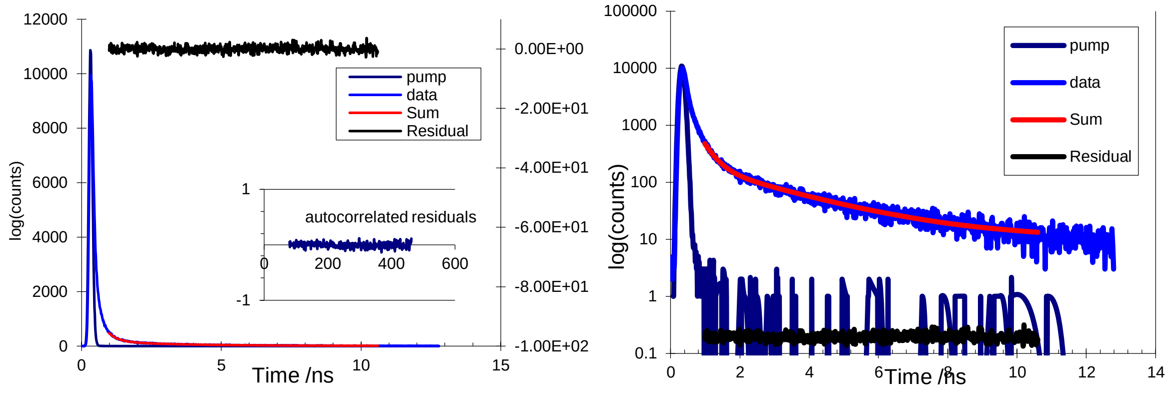

Figure S13 Lifetime decay for **Gd.1** at 410 nm (ex = 296 nm)

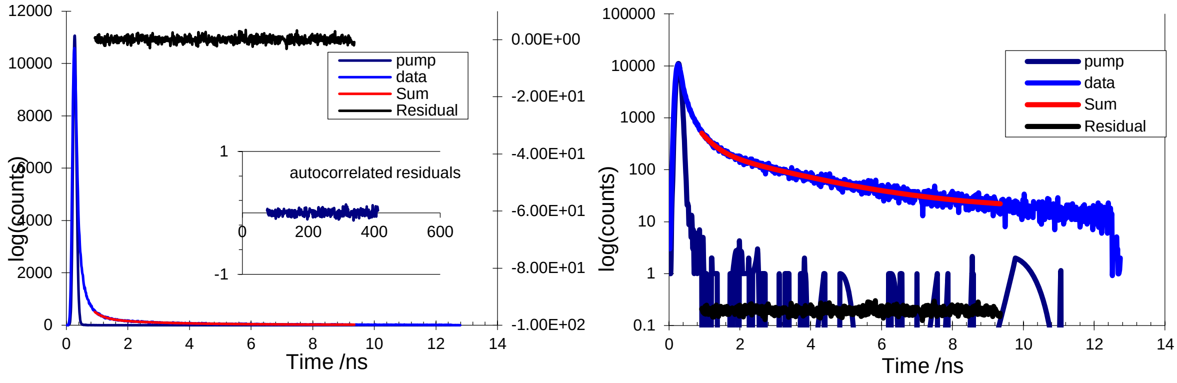

Figure S14 Lifetime decay for **Gd.1 Zn** at 410 nm (ex = 296 nm)

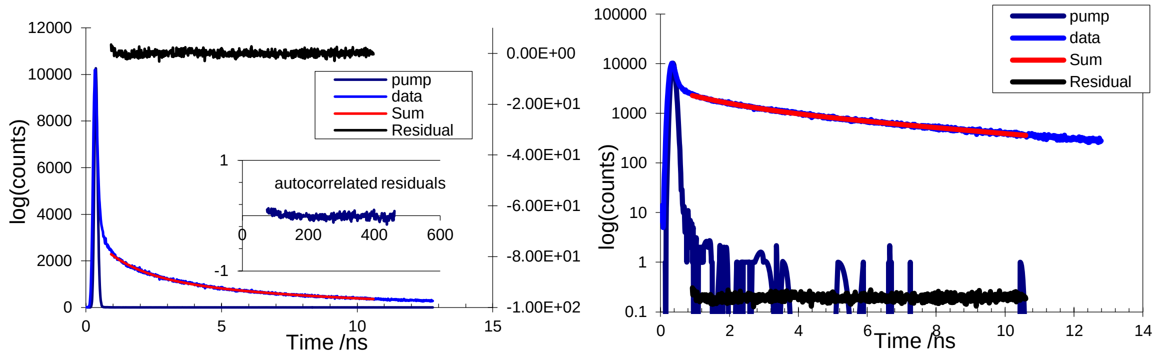

Figure S15 Lifetime decay for **Eu.1 Zn** at 500 nm (ex = 296 nm)

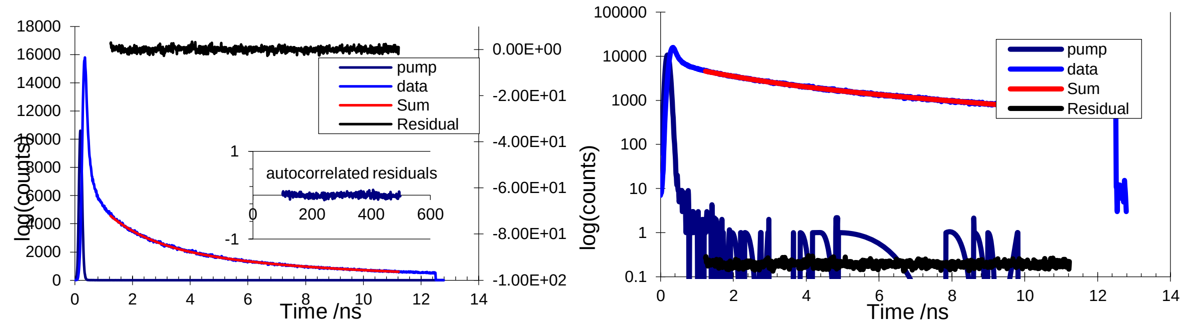

Figure S16 Lifetime decay for **Gd.1 Zn** at 500 nm (ex = 296 nm)

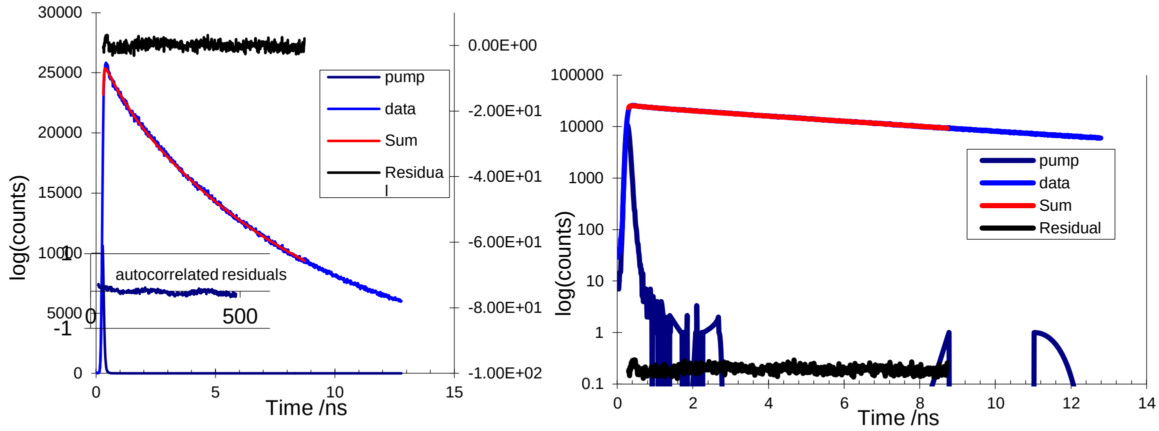

Figure S17 Lifetime decay for **Gd.1 Zn** at 500 nm (ex = 371 nm)

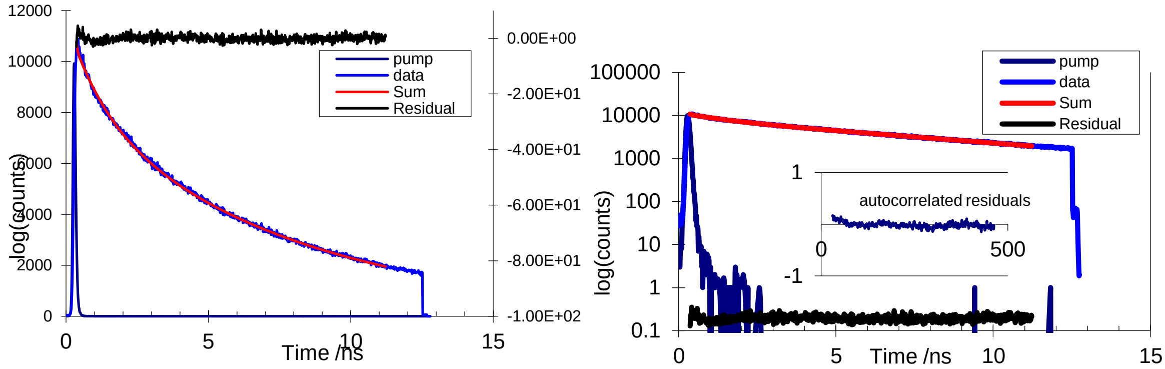

Figure S18 Lifetime decay for **Eu.1 Zn** at 500 nm (ex = 371 nm)

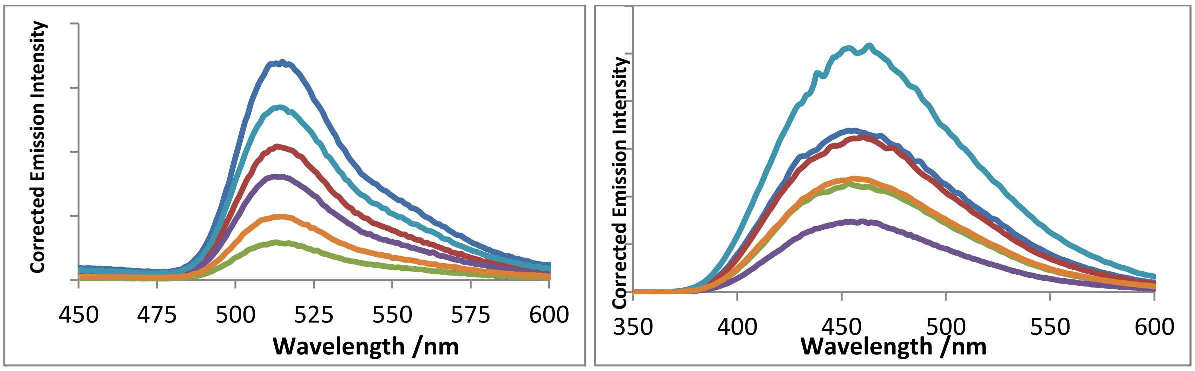

Figure S19 Quantum yield measurements; standards are fluorescein (left) and quinine (right)

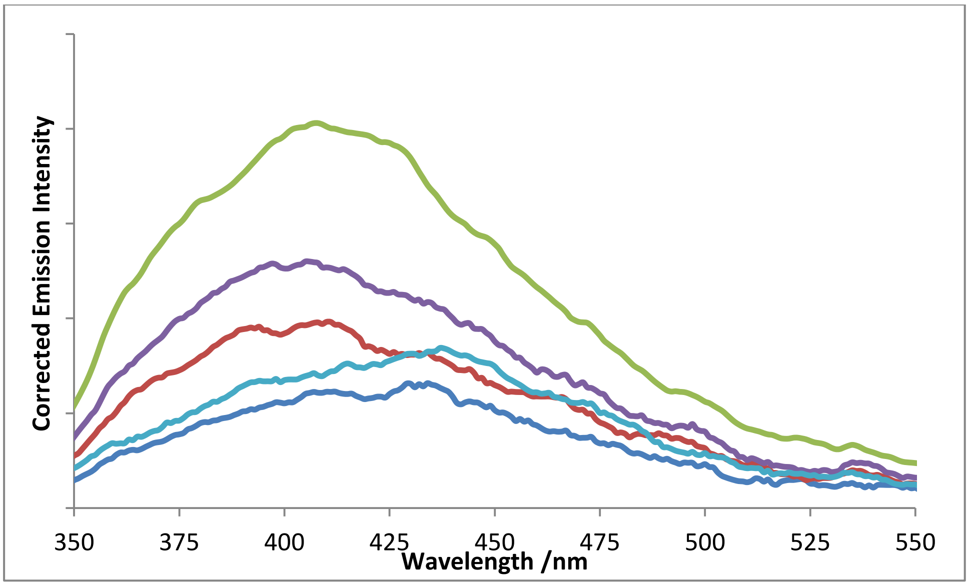

Figure S20 (a) Quantum yield measurements for **Eu.1**

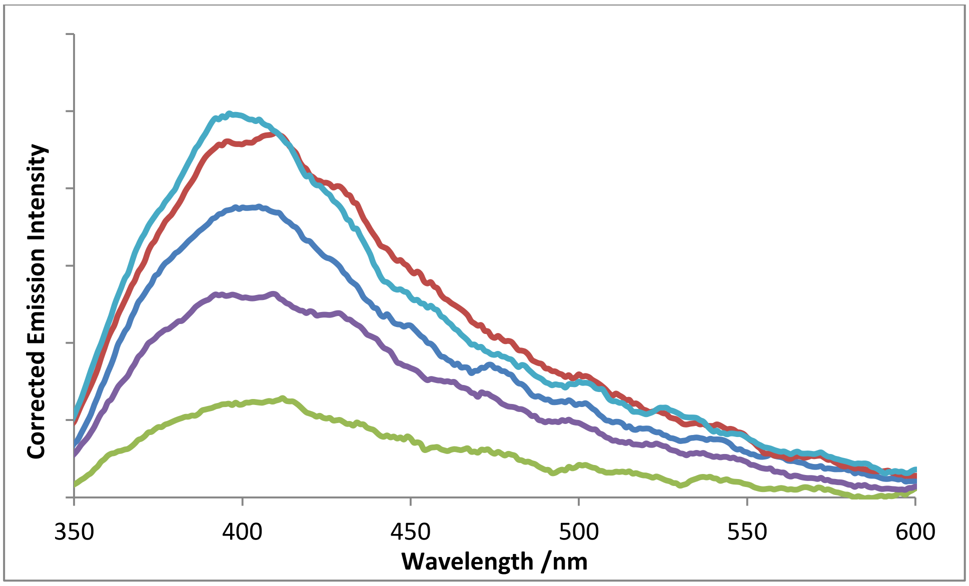

Figure S20 (b) Quantum yield measurements for **Gd.1**

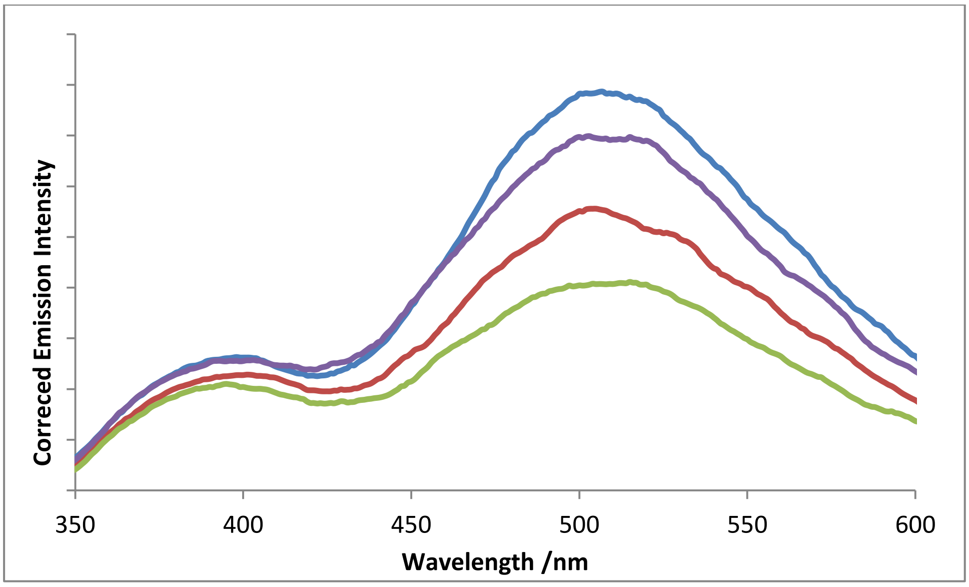

Figure S20 Quantum yield measurements for **Gd.1.Zn**

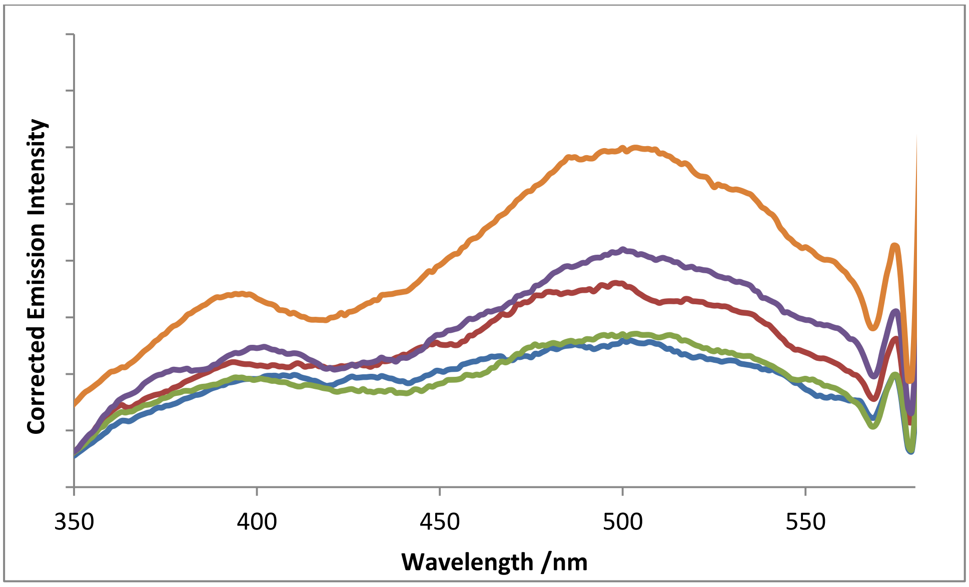

Figure S21 Quantum yield measurements for **Eu.1.Zn**

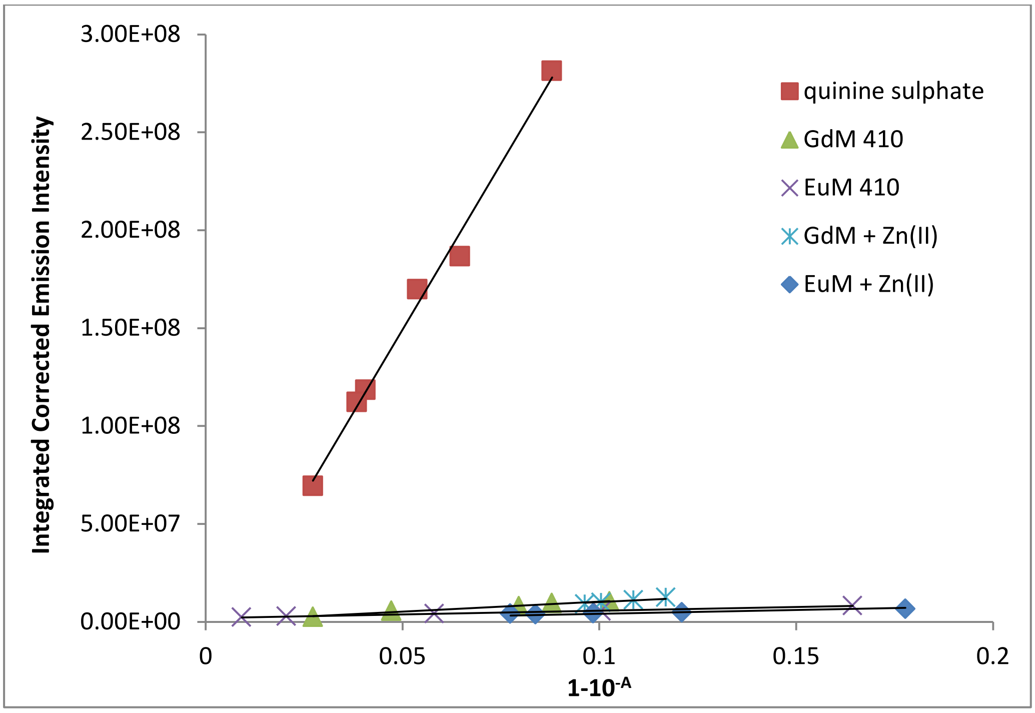

Figure S22 Quantum yield measurements (regressions for 410 nm peaks)

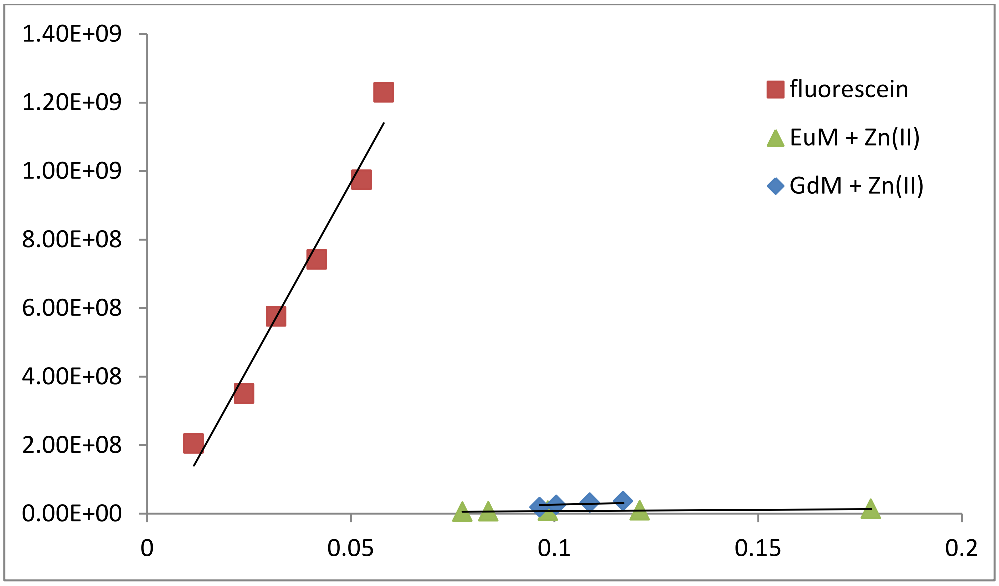

Figure S23 Quantum yield measurements (regressions for 500 nm peaks)

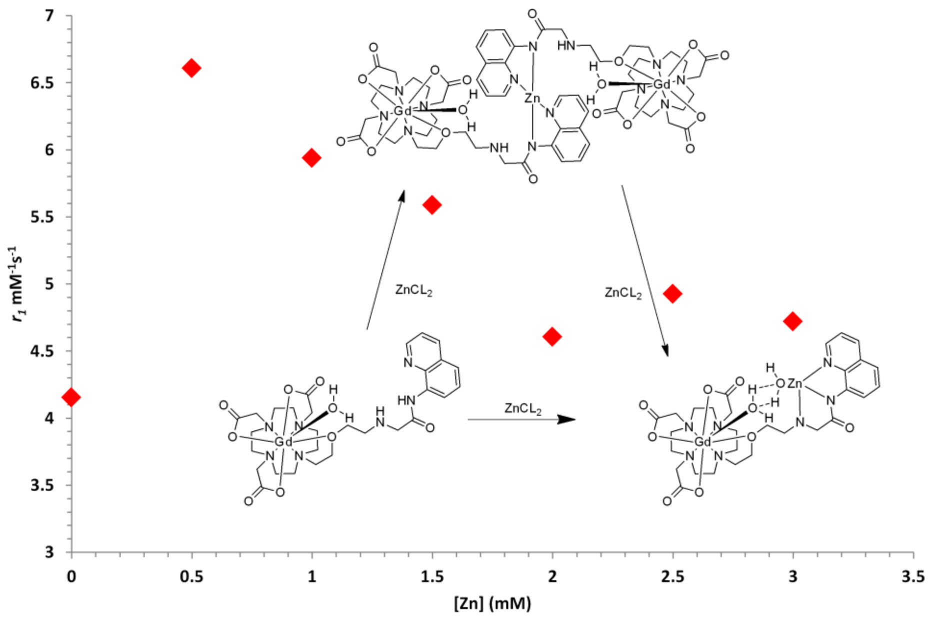

Figure S24 Gd.**1** *vs* Zn relaxation titration (1 mM, pH 7.4, 298 K)

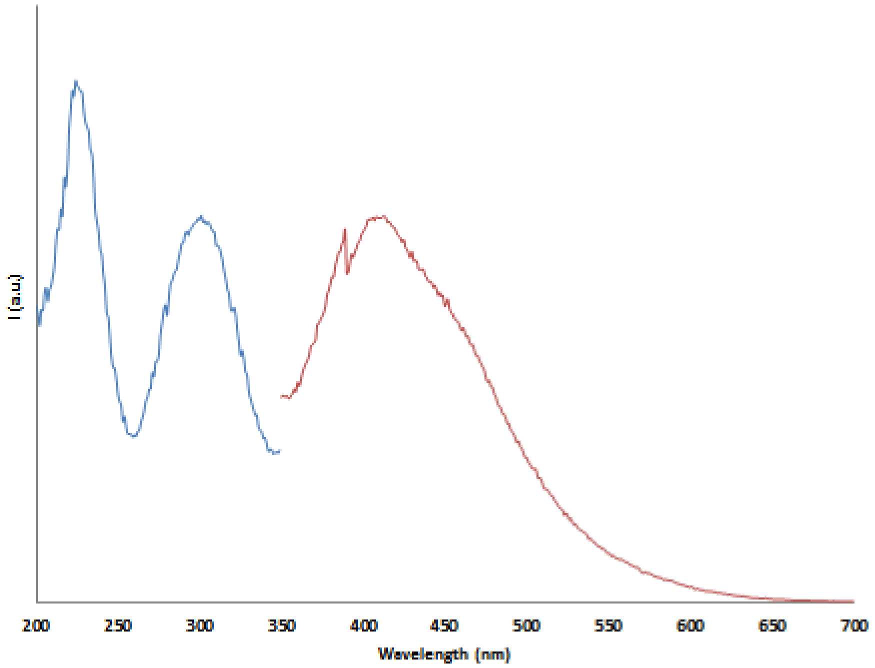

Figure S25 Gd.1 *vs* Cu fluorescence, λ_{ex} = 350 nm, 0.1 ms delay (1 mM, Cu 5mM pH 7.4, 298 K)

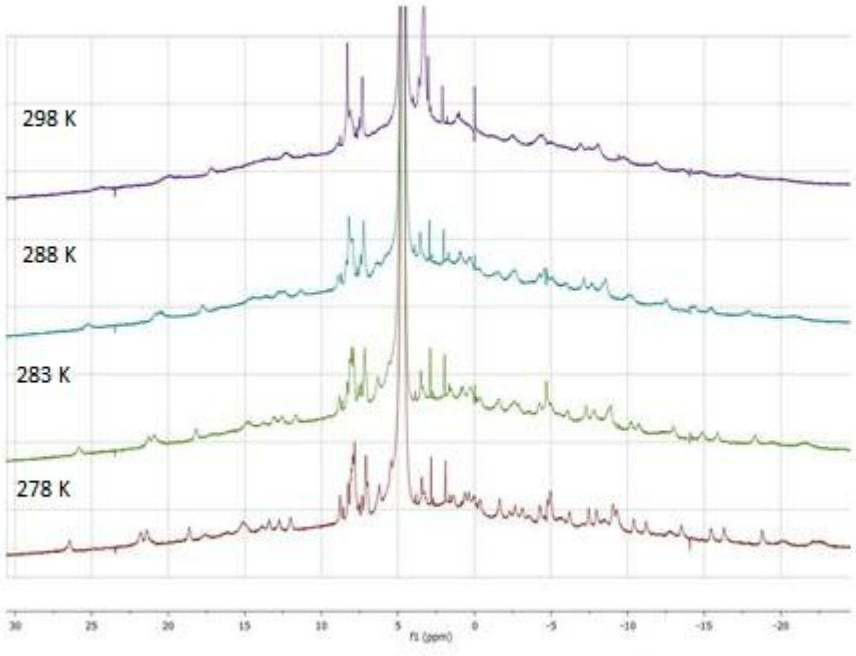

Figure S26 ^{1}H NMR spectrum of **Eu.1** in D_2O, showing definition in the ring protons around the cyclen with variable temperature.

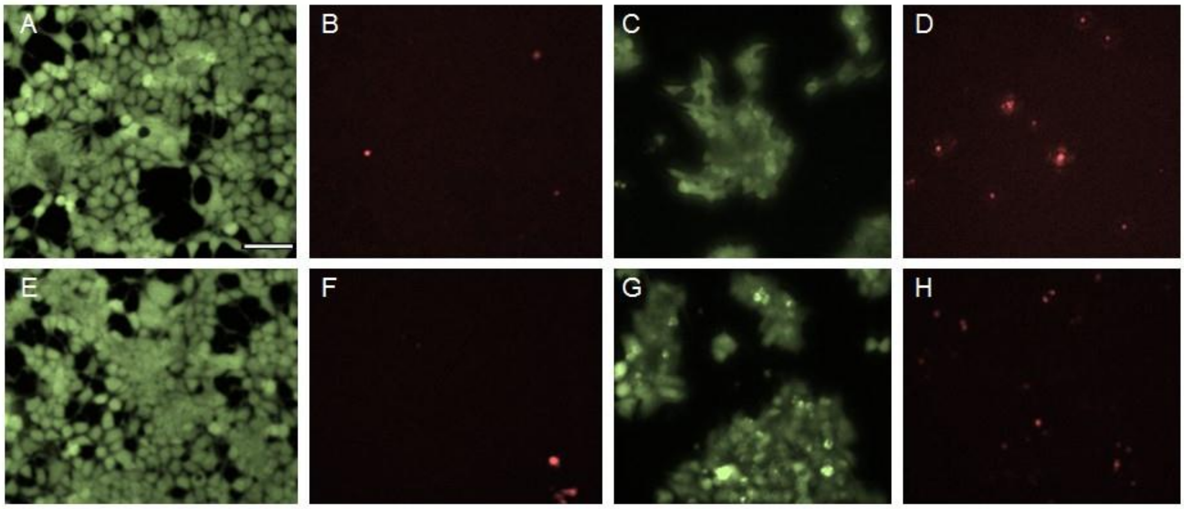

Figure S27. Representative images of the Gd.1 viability assay. Cells were incubated with KREBS media (A-D) or KREBS with 50 µM **Gd.1** (E-H) for one hour, washed twice in KREBS buffer, and stained with Calcein AM (1.5 µM, green) and propidium iodide (2.5 µM, red) for 10 minutes. The **Gd.1** incubated HEK cells (E, F) were not significantly different in viability to the control HEK cells (A, B). The **Gd.1** incubated MIN6 cells (G, H) were not significantly different in viability to the control MIN6 cells (C, D). Scalebar: 50 µm.

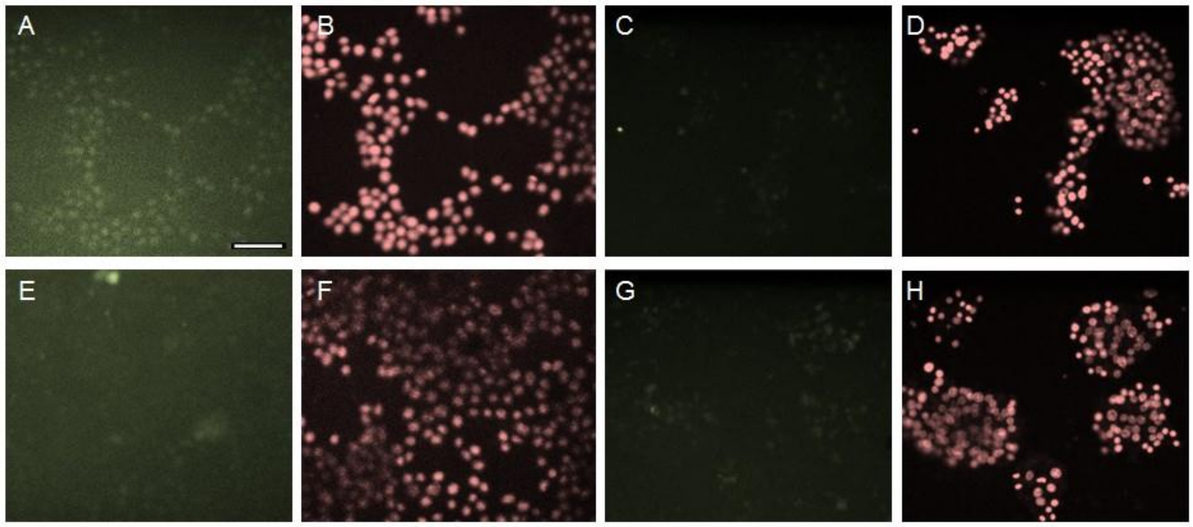

Figure S28. Representative images of the control cells killed by Triton X-100. Cells were incubated with KREBS media (A-D) or KREBS with 50µM **Gd.1** (E-H) for one hour, washed twice in KREBS buffer, and stained with Calcein AM (1.5 µM, green) and propidium iodide (2.5 µM, red) for 10 minutes. 0.2% Triton X-100 was added to each well, and cells were visualized. The **Gd.1** incubated HEK cells (E, F) and the control HEK cells (A, B) were killed with over 99% efficiency, whereas the **Gd.1** incubated MIN6 cells (G, H) and the control MIN6 cells (C, D) were killed with over 95% efficiency, with live cells being found in the centre of cell clusters. Scalebar: 50 µm.

The numbers for the Gd-mono viability assay

The numbers for the non-killed cells are:
Non-stained HEK cells alive: 99.57%
Gd.1 stained HEK cells alive: 99.42%
Non-stained MIN6 cells alive: 90.88%
Gd.1 stained MIN6 cells alive: 91.87%

And triton killed cells:
Non-stained HEK cells alive: 0%
Gd.1 stained HEK cells alive: 0.86%
Non-stained MIN6 cells alive: 2.08%
Gd.1 stained MIN6 cells alive: 4.55%

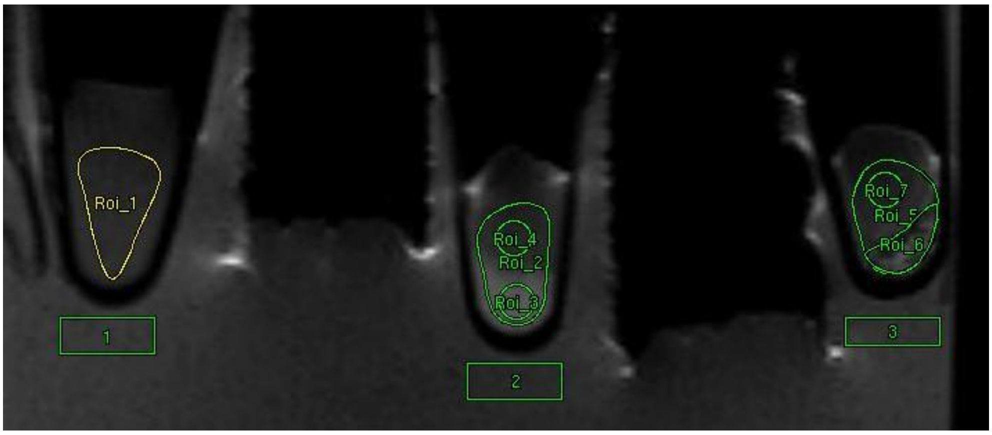

Figure S29. MRI image of Islet cells incubated with **Gd.1**. 1 represents water, 2 represents islet cells with no **Gd.1** and 3 islet cells incubated with **Gd.1** (4.7T 298K)

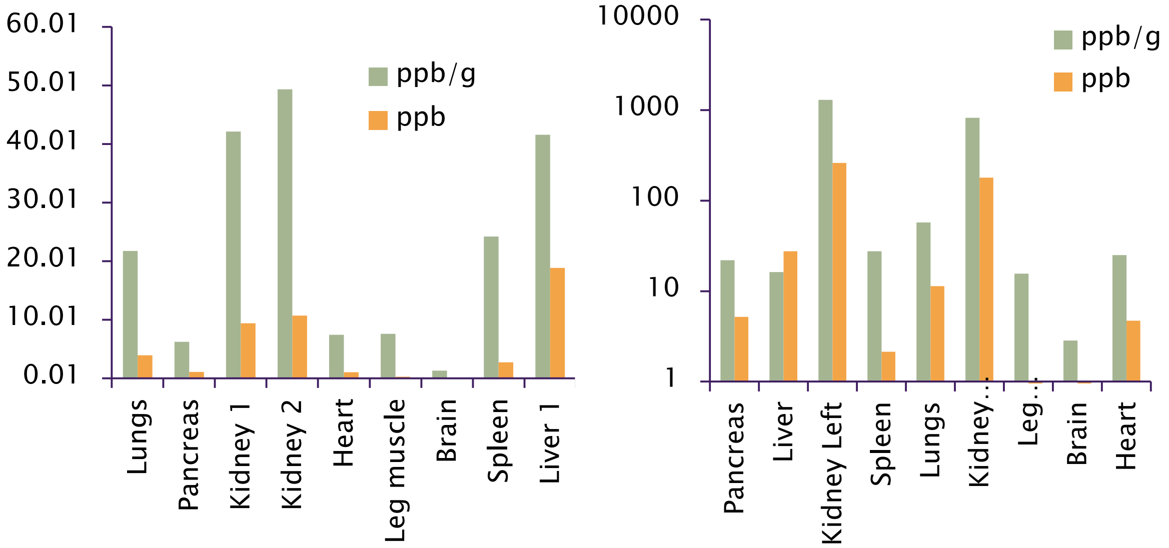

Figure S30 Biodistribution studies, 200 ug of **Gd.1** intravenously injected via tail vein. 30 minutes after injection on the left and 180 minutes after injection on the right. Organs dissolved in nitric acid and ICPMS to give gadolinium concentration.

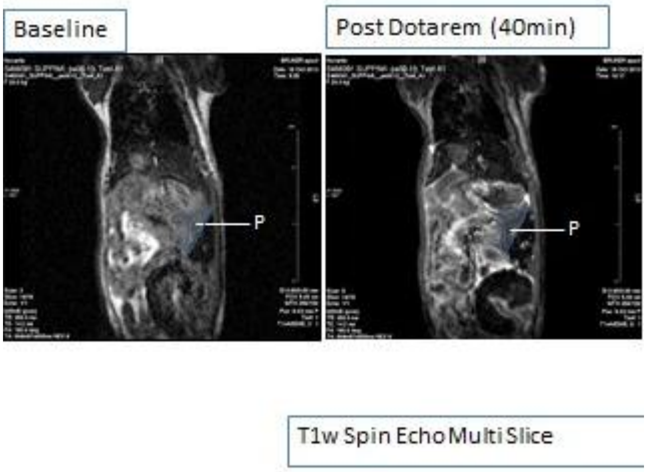

Figure S31 T1w spin echo MRI images of mouse intravenously injected with **Gd.DOTA** (0.1 mmol/kg)

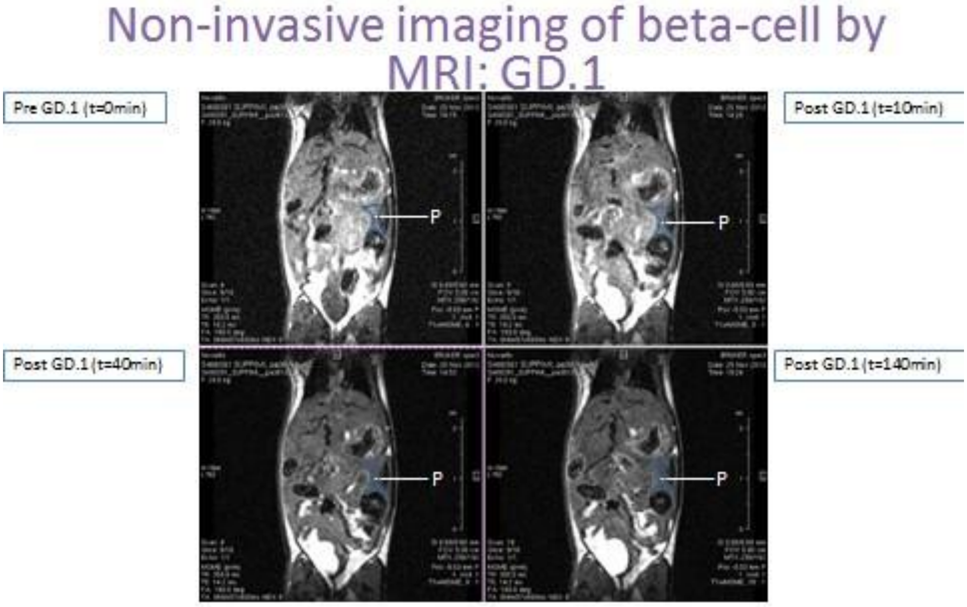

Figure S32 T1w spin echo MRI images of mouse intravenously injected with **Gd.1** (0.1 mmol/kg)

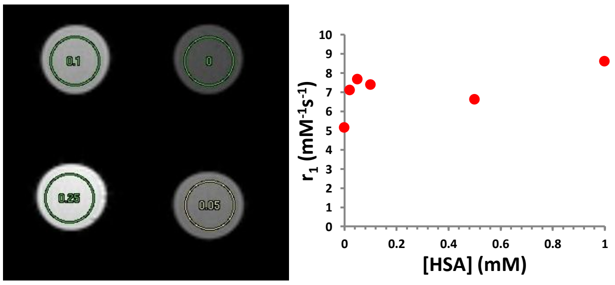

Figure S33 A) MRI phantoms of **Gd.1** (0, 0.05, 0.1 and 0.25 mM) (pH 7.4, 4.7T, 298 K), B) **Gd.1** vs Human Serum Albumin (HSA), binding (pH 7.4, 298 K and 4.7T).

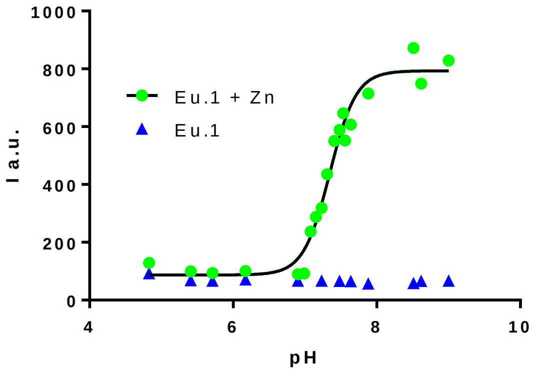

Figure S34 Eu.1 *vs* pH fluorescence titration, with zinc (green circles) and without zinc (blue triangles), λ_{ex} = 325 nm, λ_{em} = 510 nm (1 mM, 298 K, 0.1M KCl)

S1 Tsuboi T, da Silva Xavier G, Leclerc I, Rutter GA. *J Biol Chem. 26* **2003** 26 52042-52051.

S2 Beeby A.; Clarkson, I. M.; Dickins, R. S.; Faulkner, S.; Parker, D.; Royle L.; de Sousa, S. A.; Williams, J. A. G.; Woods, M. *J. Chem. Soc. Perkin Trans. 2* **1999**, 493-503.
